# AIDCare: A Technical Proof-of-Concept for Integrating Personalized Nutrition, Physical Activity, and AI-Assisted Care in Diabetes Lifestyle Management

**DOI:** 10.3390/nu18203353

**Published:** 2026-10-13

**Authors:** Adnan Kavak, Muhammad Jamil, Halil Bahadır Arın, Alpaslan Burak İnner, Hossein Fotouhi, Emre Gezer, Özlem Alkan, Zeynep Cantürk, Alev Selek, Gautam Srivastava

**Affiliations:** 1 Department of Computer Engineering, Kocaeli University, İzmit 41001, Kocaeli, Türkiye; jamil138.amin@gmail.com (M.J.); bhdrarin@gmail.com (H.B.A.); binner@kocaeli.edu.tr (A.B.İ.); 2 Wireless Information and Intelligent Systems (WINS) Research Center, Kocaeli University, İzmit 41001, Kocaeli, Türkiye; 3 Akatech Information and Health Technologies Ltd., Kocaeli University Technopark, Başiskele 41275, Kocaeli, Türkiye; 4 Department of Computer Science and Engineering, Mälardalen University (MDU), 72123 Västerås, Sweden; 5 Department of Endocrinology and Metabolism Diseases, Kocaeli University, Umuttepe, İzmit 41001, Kocaeli, Türkiye; emre.gezer@kocaeli.edu.tr (E.G.); md.ozlemalkan@gmail.com (Ö.A.); zeynepcanturk@kocaeli.edu.tr (Z.C.); alev.selek@kocaeli.edu.tr (A.S.); 6 Department of Computer Science and Engineering, Sogang University, Seoul 04107, Republic of Korea; 7 Research Center for Interneural Computing, China Medical University, Taichung 40402, Taiwan; 8 Centre for Research Impact & Outcome, Chitkara University Institute of Engineering and Technology, Chitkara University, Rajpura 140401, Punjab, India

**Keywords:** AIDCare, diabetes lifestyle management, personalized nutrition, physical activity monitoring, mobile health (mHealth), Internet of Medical Things (IoMT), Medical Large Language Models, clinician in the loop

## Abstract

**Background/Objectives:** Medical nutrition therapy and structured lifestyle self-management are fundamental to diabetes care, yet conventional mobile health tools typically track diet, physical activity, and glucose measurements in isolation, with limited connection to clinical oversight. This study presents AIDCare, an integrated mobile health platform that connects camera-based nutritional assessment, wearable physical activity tracking, physiological monitoring, and clinician-supervised conversational artificial intelligence (AI) to support personalized dietary and lifestyle self-management in diabetes. **Methods:** AIDCare links a patient mobile application with wearable sensors, backend analytical services, and an interactive clinician portal. Dietary assessment uses smartphone camera imaging to identify foods and estimate meal contents, specifically carbohydrates, protein, and dietary fiber, against nutritional reference data. Physical activity and energy expenditure are captured through smartphone and smartwatch motion sensors. To support contextual lifestyle guidance, an open-access medical conversational language model was adapted using 24,612 structured digital health and diabetes educational articles. A clinician-in-the-loop mechanism allows healthcare providers to inspect AI-generated recommendations and adjust personalization parameters. System feasibility was evaluated through benchtop and system-level testing without patient recruitment, using synthetic patient profiles across 400 data-acquisition pipeline trials and an intent-classification benchmark spanning nutrition, activity, glucose monitoring, and physician consultation. **Results:** Across the 400 software-pipeline trials, the platform achieved data acquisition, transmission, and processing completion rates of 97.5%, 96.5%, and 95.5%, respectively, with backend response times under 1.8 s. In the recommendation-intent benchmark, the medical language model achieved 81.0% accuracy, 83.5% precision, 79.0% recall, and an F1-score of 81.2%. The platform successfully linked image-based meal nutrient estimates (carbohydrate, protein, and dietary fiber) with physical activity metrics and physiological tracking within a unified interface under clinician supervision. **Conclusions:** AIDCare establishes the technical feasibility of a unified digital platform that integrates dietary nutrient estimation, physical activity tracking, and clinician-guided AI decision support for diabetes self-management. By bridging daily nutritional intake with metabolic tracking and professional oversight, the architecture provides a functional framework for personalized lifestyle care. Prospective clinical trials in patient cohorts are required to establish nutritional estimation accuracy against weighed food records, long-term user adherence, and clinical efficacy on glycemic control.

## 1. Introduction

Diabetes is a major chronic metabolic disorder that requires continuous monitoring and day-to-day self-management beyond conventional clinical encounters. The condition encompasses several forms with distinct pathophysiological mechanisms and operational nutritional pathways: Type 1 diabetes involves autoimmune beta-cell destruction, requiring meticulous carbohydrate counting to determine prandial insulin boluses using individualized Insulin-to-Carbohydrate Ratios (ICR) and Insulin Sensitivity Factors (ISF); Type 2 diabetes is characterized by insulin resistance and progressive secretory defect, where Medical Nutrition Therapy (MNT) prioritizes caloric moderation, dietary fiber intake, low glycemic load, and weight management, with basal or prandial insulin introduced when oral agents are insufficient; and Gestational Diabetes Mellitus (GDM) involves pregnancy-induced hormonal insulin resistance necessitating strict postprandial glycemic thresholds and controlled carbohydrate distribution across meals [1,2,3,4,5]. Dietary carbohydrates represent the primary macronutrient driving immediate postprandial blood glucose excursions, while co-ingested dietary fiber and proteins retard gastric emptying and intestinal absorption, attenuating glycemic spikes and postprandial glycemic variability. Consequently, effective diabetes care demands continuous, coordinated attention to dietary macronutrient intake, physical activity energy expenditure, and physiological status within everyday environments [6,7].

Despite the centrality of nutrition, accurate dietary assessment remains one of the most formidable challenges in diabetes self-management [8,9,10,11]. Conventional dietary monitoring methods—such as paper-based food records, 24-h recalls, and retrospective questionnaires—are notoriously vulnerable to recall bias, portion-size misestimation, and underreporting of up to 50% of caloric and carbohydrate intake when compared against objective reference biomarkers such as doubly labelled water or weighed duplicate meals [12]. The widespread availability of smartphones offers an opportunity to advance digital dietary assessment through computer-vision-based food logging [13]. Camera-based dietary assessment can identify food items, segment portions, and estimate meal macronutrients (carbohydrates, protein, and dietary fiber), transforming meal photography into structured nutritional telemetry. However, image-based nutrition estimation faces major practical hurdles, including visual occlusion in layered foods, mixed composite dishes, hidden fats, and monocular depth ambiguities, with contemporary vision algorithms demonstrating Mean Absolute Percentage Errors (MAPE) between 14% and 25% for carbohydrate estimation on benchmark datasets [9,10].

Furthermore, nutritional intake does not act in isolation. A given meal produces disparate postprandial glycemic responses depending on concurrent physical activity, baseline glucose levels, and individual metabolic factors. Wearable devices and connected health sensors can continuously capture heart rate, physical activity, and active energy expenditure, while digital glucometers provide physiological feedback [14,15]. Existing mobile health (mHealth) applications remain largely fragmented, isolating dietary calorie counters from physical activity trackers and physiological logs [16,17,18]. Most commercially available nutrition apps lack real-time physiological feedback, fail to convert dietary metrics into clinical decision support (such as carbohydrate-to-insulin calculations), and provide no mechanism for professional clinical oversight [19,20,21]. There is a critical need for an integrated platform that bridges image-based nutritional assessment with wearable sensor telemetry and clinician-guided artificial intelligence (AI).

To address these challenges, AIDCare, an integrated multimodal mHealth platform designed to support personalized nutrition, physical activity, and lifestyle management in diabetes, is proposed. The architecture unifies image-based dietary assessment (carbohydrate, protein, and dietary fiber estimation), physical activity tracking, physiological monitoring, personalized decision support, and clinician supervision. Smartphone-based camera and motion inputs are combined with connected smartwatch telemetry through the AIDCare mobile application and backend infrastructure. The platform incorporates analytical modules for vital measurement processing, logistic HbA1c risk prediction, carbohydrate-based insulin bolus calculation, nutrition and diet estimation, and active energy expenditure assessment. In addition, an open-access Medical Large Language Model (MedGemma 4B-IT [22]) was adapted via parameter-efficient fine-tuning (QLoRA) using 24,612 structured health and educational articles, while an AI-powered personalized recommender integrates multimodal inputs with patient goals. A clinician-in-the-loop dashboard enables healthcare providers to review AI-generated responses and calibrate recommendation parameters.

The primary objective of this study is to design and evaluate the technical feasibility and system-level architecture of AIDCare as a proof-of-concept platform for personalized nutrition and lifestyle diabetes management. Rather than evaluating individual sensing or nutritional algorithms in isolation, this study investigates their end-to-end integration across data acquisition, transmission, analytical processing, and clinician oversight using synthetic patient profiles and controlled software-pipeline trials. The main contributions of this study are as follows:

1. An integrated multimodal mHealth architecture links smartphone camera-based dietary assessment for carbohydrate, protein, and fiber estimation with wearable physical activity tracking and physiological monitoring to support comprehensive diabetes lifestyle management.
2. An end-to-end data pipeline transfers heterogeneous dietary, motion, and vital measurements to backend services for structured normalization and multi-domain health analysis.
3. Analytical modules for vital sign smoothing, HbA1c risk assessment, nutritional macronutrient quantification, active energy expenditure calculation, and carbohydrate-informed insulin bolus decision support are integrated within a single coordinated framework.
4. An open-access Medical LLM adapted on 24,612 structured educational articles and an adaptive multi-objective recommender are integrated to provide contextual lifestyle and nutritional guidance aligned with patient-specific goals.
5. A clinician-in-the-loop mechanism enables healthcare professionals to supervise AI recommendations, adjust personalization weights, and oversee nutritional and therapeutic targets.
6. The technical feasibility and pipeline reliability of the integrated architecture are demonstrated through 400 multimodal data-acquisition trials and recommendation intent-classification benchmarking.

The significance of this work lies in connecting dietary nutritional assessment, physical activity, and physiological tracking into a cohesive clinical and self-management workflow, moving beyond disconnected consumer calorie trackers. By establishing an automated assessment-to-personalization pathway under clinical supervision, AIDCare provides a technical foundation for precision nutrition and lifestyle support in everyday settings. Because the present investigation focuses on architectural implementation and technical feasibility using synthetic profiles, clinical accuracy against weighed food records, behavioral engagement, and long-term metabolic efficacy remain subjects for future prospective clinical trials.

## 2. Related Work

### 2.1. mHealth Platforms for Diabetes Self-Management

The proliferation of smartphones has resulted in a large number of mHealth applications intended to support diabetes self-management. Existing applications provide functions such as blood glucose recording, dietary and carbohydrate logging, physical activity tracking, medical resources, medication management, and progress monitoring, although these functions are not consistently integrated within a single platform [23]. Despite these developments, existing mHealth platforms have reported limitations related to usability, engagement, data quality, and personalization [24]. Manual entry remains an important component of many applications, which can increase user burden and introduce errors, particularly for complex information such as dietary intake. Usability challenges can further restrict adoption, particularly among users with lower digital literacy or those who may find frequent data entry inconvenient [25].

These limitations indicate that the next generation of diabetes mHealth systems should move beyond the traditional model of manual data logging and static information delivery. In particular, digital support should connect dietary intake, physical activity, physiological information, and patient-specific goals within a coherent self-management workflow. Smartphone-based information acquisition can reduce dependence on manual input for selected categories of information, while wearable and IoMT devices can provide complementary physiological and activity-related measurements. However, these data sources must be integrated with appropriate analytical and personalization mechanisms to generate meaningful health-management support. Therefore, data acquisition, processing, intelligent interpretation, and personalized interaction should be considered as interconnected components of a complete digital health architecture.

A comparative analysis of basic and advanced features of leading diabetes-specific digital health platforms—including mySugr [26], Glooko [27], One Drop [28], and LibreView [29]—alongside the proposed AIDCare architecture is presented in Table 1. To ensure an objective and clinically grounded comparison, system capabilities are explicitly categorized into fully implemented and operational functions (√), architected or preliminary prototypes (∘), and unsupported functions (×). This distinguishes AIDCare’s realized software contributions from its conceptual modules while contrasting them with commercially established diabetes care platforms.

### 2.2. Digital Nutrition, Physical Activity, and IoMT in Diabetes Management

Diabetes represents a primary clinical domain where digital nutrition and precision lifestyle interventions can directly improve metabolic outcomes. Medical nutrition therapy requires rigorous consideration of meal composition, macronutrient distribution, and glycemic impact [30]. Traditional dietary assessment methodologies, such as paper-based food frequency questionnaires (FFQs) and 24-h recalls, exhibit recognized limitations in clinical practice, including substantial recall bias, deliberate underreporting of calorie-dense items, and inaccurate portion estimation. These deficiencies are particularly detrimental in diabetes care, where precise estimation of carbohydrate mass and meal composition directly governs postprandial glucose dynamics and prandial insulin dosing [31].

Recent advances in digital nutrition have sought to mitigate these barriers through smartphone-enabled image-based dietary assessment and sensor integration [32]. Mobile camera systems can capture meal scenes, apply computer-vision algorithms for food item segmentation and volumetric approximation, and map detected items to nutritional databases for automated macronutrient estimation (carbohydrates, protein, and dietary fiber) [9,10]. Furthermore, wearable activity monitors and continuous physiological sensors provide real-time streams of movement intensity and energy expenditure [33]. However, existing digital nutrition systems typically operate in isolation: commercial diet tracking apps rarely interface with clinical decision-support algorithms, while diabetes management devices often overlook meal composition nuances [34]. Connecting image-based nutritional estimation with continuous physiological feedback and clinical oversight addresses this critical disconnect, advancing the frontier of precision nutrition in chronic disease care.

Because the dietary assessment module within AIDCare builds upon our previous foundational work in *Nutrients* [9,10], it is critical to clarify which elements are inherited and which represent newly introduced contributions:

- **Inherited Elements:** AIDCare inherits the core computer-vision methodologies established in [9,10], specifically the deep-learning segmentation models (food vs. non-food masking and multi-class item identification) and monocular volumetric approximation algorithms that estimate food volume from smartphone imagery.
- **Newly Introduced Elements:** In contrast to our earlier work—which functioned strictly as standalone computer-vision benchmarks for single-meal food classification and carbohydrate estimation—AIDCare introduces substantial architectural and functional expansions: (i) performing multi-nutrient biochemical profiling that simultaneously quantifies protein, dietary fiber, and net carbohydrates (${\mathrm{CHO}}_{\mathrm{net}}$) mapped to national nutritional reference tables (USDA and TÜRKOMP); (ii) coupling meal telemetry with continuous wearable accelerometer signals to compute active energy expenditure (${\mathrm{EE}}_{\mathrm{act}}$) and contextualize postprandial glucose dynamics; (iii) direct feeding confirmed meal carbohydrates into a clinician-supervised prandial insulin bolus calculator with automated hypoglycemia lockouts and active insulin-on-board subtraction; and (iv) integration into an end-to-end mHealth architecture featuring an open-access Medical LLM (MedGemma 4B-IT QLoRA) and a bidirectional Clinician Dashboard for dynamic recommendation weight adaptation.

### 2.3. Multimodal Data Integration for Personalized Healthcare

The integration of multiple information sources has become increasingly important in digital healthcare because individual data sources provide only partial representations of a patient’s health state. Multimodal healthcare systems can combine physiological measurements, activity information, dietary information, patient-reported data, and other contextual signals to construct a more comprehensive patient representation [35]. In mobile environments, this may involve combining smartphone information with wearable measurements and user-generated inputs.

Multimodal integration also introduces several technical challenges. Different devices and information sources may generate heterogeneous data at different temporal resolutions, formats, and levels of reliability. Sensor-derived measurements may require preprocessing, normalization, and contextual interpretation before they can be used by higher-level analytical modules [36]. Furthermore, patient-generated information obtained through cameras, microphones, or manual inputs may be unstructured and require additional processing before integration with structured physiological measurements.

For diabetes self-management, multimodal integration is particularly relevant because dietary behavior, physical activity, physiological measurements, and patient interactions are interdependent [37]. A system that integrates these sources can potentially provide a richer representation of the patient’s daily context than a platform based exclusively on individual measurements. The AIDCare architecture addresses this requirement by connecting smartphone-based camera, microphone, and accelerometer inputs with smartwatch-derived information and patient-generated health data through a common mHealth and backend infrastructure.

### 2.4. Artificial Intelligence and Large Language Models in Digital Diabetes Care

Artificial intelligence has increasingly been incorporated into digital healthcare applications to support prediction, classification, recommendation, and patient interaction. In diabetes management, AI-based approaches have been investigated for glucose prediction, risk assessment, dietary analysis, activity recognition, personalized recommendations, and clinical decision support [38]. These approaches provide opportunities to transform heterogeneous health and lifestyle information into higher-level interpretations and personalized support.

More recently, large language models (LLMs) have introduced new possibilities for patient-facing healthcare applications because of their ability to process natural-language information and generate context-sensitive responses. Studies have explored LLMs for healthcare monitoring, patient communication, medical question answering, and self-management support [39]. In diabetes-related applications, LLMs may provide a flexible mechanism for interpreting patient-generated information and facilitating conversational access to health-related resources.

The use of LLMs in healthcare introduces important challenges. LLM-generated responses can contain factual errors or hallucinated information, which can be particularly problematic when users interpret generated content as medical guidance [40]. Additional concerns include transparency, reliability, privacy, contextual consistency, and appropriate human oversight [41]. Consequently, LLMs should not be considered a replacement for clinical expertise. Their integration into healthcare systems requires appropriate constraints, contextualization, and mechanisms for human review.

Within AIDCare, the Medical LLM is positioned as an intelligent interaction component within a larger health-management architecture rather than as an independent source of unrestricted medical advice. The LLM can utilize patient-specific information and conversation history to support contextualized interaction, while its outputs are integrated with structured health information, analytical modules, personalized goals, and clinician oversight. This architectural positioning allows LLM-based interaction to complement rather than replace the analytical and clinical components of the platform.

Within AIDCare, medical content is derived from [42] and is structured using the H5P [43] content organizer. The H5P content is parsed and normalized before being used for Medical LLM fine-tuning. MedGemma 4B-IT [22] was used as the base model and trained on the H5P-organized content through Quantized Low-Rank Adaptation (QLoRA) [44]. This provides a mechanism for incorporating structured medical material into the broader nutrition, lifestyle, personalization, and health-management pipeline.

### 2.5. Personalization in Digital Diabetes Self-Management

Personalization is an important requirement for chronic disease management because patients differ in their physiological characteristics, lifestyles, health knowledge, behavioral patterns, and self-management objectives. Digital healthcare systems have therefore explored several personalization approaches, ranging from predefined rules and profile-based recommendations to machine-learning-based recommender systems and adaptive AI models [45].

Rule-based approaches offer advantages in terms of transparency, consistency, and auditability because their outputs can be traced to predefined conditions or expert-defined logic [46]. However, rule-based systems can be limited when patient information is complex, heterogeneous, or difficult to represent through predefined conditions. Conversely, machine-learning and recommender-system approaches can provide greater adaptability but may require substantial data and can introduce challenges related to interpretability and generalization [45,47].

LLM-based approaches offer an additional mechanism for personalization by allowing heterogeneous and unstructured patient information to be interpreted through natural-language interaction. However, their probabilistic nature makes unrestricted use inappropriate for safety-sensitive healthcare applications. The AIDCare architecture therefore adopts a broader personalization strategy in which structured health information, nutrition-related information, physical activity information, patient-specific goals, analytical outputs, conversation history, and clinician oversight are integrated into the recommendation and interaction process. This approach emphasizes contextual personalization within an integrated healthcare architecture rather than relying exclusively on a single recommendation technique.

### 2.6. Clinician-in-the-Loop-Based Digital Healthcare Systems

Although automated AI systems can provide scalable digital support, healthcare applications require appropriate mechanisms for human oversight. Clinician-in-the-loop approaches incorporate healthcare professionals into the AI-assisted workflow to review outputs, correct inappropriate responses, and provide additional clinical context [48]. Such mechanisms are particularly important when AI systems process patient-specific information or generate recommendations that may influence health-related decisions.

The integration of clinician oversight can also improve the alignment between automated personalization and individualized care. Rather than treating AI-generated outputs as final decisions, clinician-in-the-loop architectures can establish a feedback pathway in which AI systems provide computational support while healthcare professionals retain an appropriate supervisory role [49,50]. This principle is reinforced by recent advances in digital healthcare coordination systems. Notably, Gackowski et al. [51] demonstrated the operational necessity and clinical value of an AI-based coordination platform (the CORE system) designed to connect patients and medical staff for test result processing, longitudinal record sharing, and Individual Patient Care Plans (IPOM). In chronic disease management, bidirectional digital communication between patients and clinical providers is essential to bridge the gap between daily lifestyle self-monitoring and professional therapeutic governance. This principle is incorporated into AIDCare through the Clinician-in-the-Loop module and Clinician Dashboard. The architecture allows clinicians to review and rectify Medical LLM responses and manage patient-specific goals and treatment-related information. This component distinguishes the proposed framework from purely consumer-oriented mobile applications by explicitly incorporating professional oversight into the personalized health-management pipeline.

### 2.7. Identified Research Gaps and Architectural Objectives

The reviewed literature demonstrates substantial progress in diabetes mHealth tools, digital dietary assessment, wearable sensing, and conversational AI. However, several critical architectural and clinical translation gaps persist across existing platforms:

- **Isolated Dietary and Physical Activity Tracking:** Although commercial tools facilitate calorie counting and step tracking, dietary logging is rarely coordinated with energy expenditure, metabolic context, or prandial insulin decision support [9,23,24].
- **Sensor Fragmentation and Lack of Interoperability:** Physiological telemetry, wearable motion streams, and image-based meal captures are routinely gathered across disparate proprietary applications, hindering the generation of a cohesive longitudinal patient state [16,17,25].
- **Absence of Clinical Decision Support from Telemetry:** Acquired sensor and dietary streams are seldom synthesized into actionable clinical telemetry (e.g., carbohydrate-to-insulin calculations, glycemic smoothing, or individualized nutrition targets) [3,18].
- **Unguarded Conversational Generative AI:** While large language models present promising conversational modalities for lifestyle guidance, unconstrained consumer LLMs exhibit risks of medical hallucination, requiring rigid clinical guardrails and domain-specific grounding [48,49].
- **Omission of Clinician-in-the-Loop Supervision:** Most digital diabetes applications operate as closed consumer silos without bidirectional communication channels that allow healthcare providers to verify automated lifestyle guidance, review meal logs, or tune algorithmic personalization parameters [48,50].

To address these architectural challenges, AIDCare is designed as an integrated proof-of-concept platform connecting camera-based macronutrient quantification, wearable motion telemetry, and clinician-supervised conversational decision support within a cohesive mHealth pipeline. Smartphone and connected IoMT devices serve as enabling infrastructure for acquiring complementary information rather than constituting the sole focus of the framework.

The present study focuses on technical development and system-level evaluation. Consequently, the reviewed gaps should not be interpreted as evidence that AIDCare has already demonstrated superior clinical effectiveness, nutritional or activity assessment accuracy, patient adherence, or long-term behavioral outcomes. These aspects require prospective evaluation with individuals living with diabetes and healthcare professionals. Nevertheless, the proposed architecture provides a technical foundation for such future studies by establishing an integrated pathway through which dietary, physical activity, physiological, and contextual information can be acquired, processed, interpreted, and incorporated into individualized diabetes self-management support.

## 3. Methods

The AIDCare platform was designed and developed as an integrated multimodal mHealth architecture to support personalized nutrition, lifestyle management, and diabetes self-management. The methodology focuses on establishing an end-to-end pathway from dietary and lifestyle information acquisition to health-data processing, nutritional and physical activity assessment, personalized interaction, recommendation, and clinician oversight. In contrast to treating nutrition assessment, lifestyle monitoring, physiological analysis, personalization, and artificial intelligence as independent functions, the proposed architecture connects these components through a common mobile application and backend infrastructure. Smartphone and IoMT-based inputs provide complementary information that can be used to characterize dietary intake, physical activity, physiological status, and individual lifestyle context. The resulting system was developed as a technical proof-of-concept and evaluated at the system level under controlled conditions rather than through prospective clinical intervention or patient outcome assessment.

### 3.1. Overall System Architecture

The AIDCare architecture consists of an interconnected mobile sensing and application layer, backend processing and data-management layer, intelligent health-analysis components, and clinician-facing functionality. The mobile layer serves as the primary interface for patients and provides access to smartphone-based sensing modalities and connected wearable devices. The backend layer manages the received patient information, application logic, content processing, and communication with the analytical and intelligent components. PostgreSQL provides persistent storage for structured patient and application data. The architecture also incorporates specialized modules for vital measurement processing, HbA1c risk prediction, insulin dosage recommendation, nutrition and diet assessment, and exercise analysis. The Medical Large Language Model (MLLM), AI Personalized Recommender, and clinician-in-the-loop components provide the higher-level interaction, personalization, and oversight functionality.

**Figure 1:**
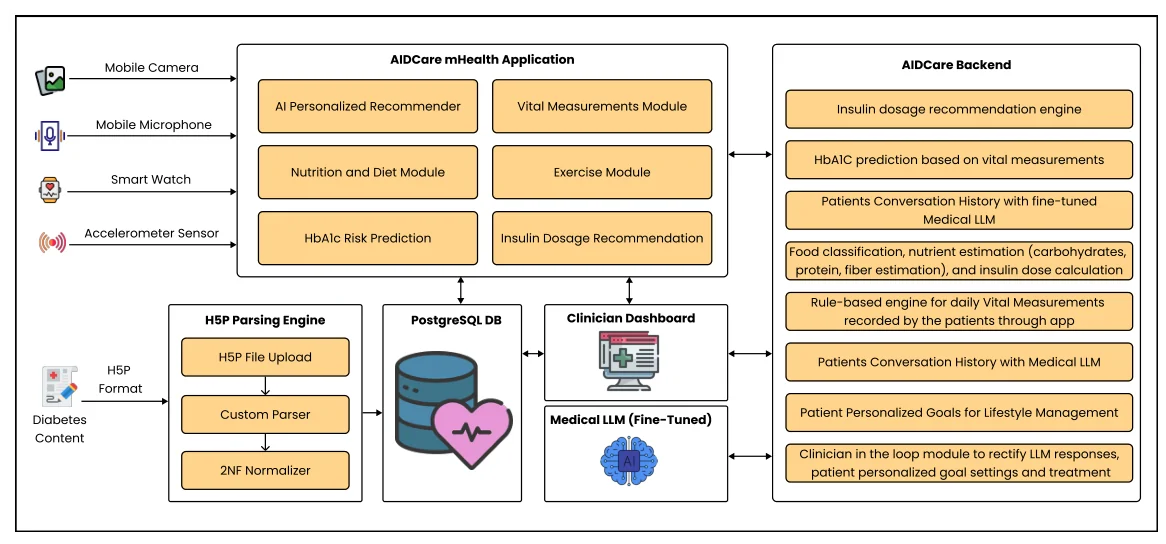
Overall architecture and data flow of the AIDCare multimodal mHealth platform.

Figure 1 presents the overall architecture and information flow of the AIDCare platform. The architecture is organized around the principle that heterogeneous patient information should first be acquired through accessible sensing and application interfaces, subsequently processed and organized by the backend, and then made available to specialized analytical and personalization components. This organization establishes a continuous pathway between patient-generated information, sensor-derived measurements, intelligent processing, and personalized health-management support.

The architecture separates data acquisition from downstream interpretation while maintaining communication between the individual components through the AIDCare backend. This separation allows heterogeneous inputs to be handled by specialized modules while preserving a common patient context. The approach also facilitates the integration of additional sensing modalities and analytical services without requiring the sensing layer itself to perform all downstream processing.

### 3.2. Multimodal Nutrition, Physical Activity, and Health Data Acquisition

The data-acquisition layer forms the foundation of the proposed AIDCare architecture by integrating nutrition-related, physical activity-related, physiological, and patient-generated information through smartphone and connected IoMT devices. The smartphone provides multiple sensing modalities, including the camera, microphone, and accelerometer, while a smartwatch provides an additional source of physiological and activity-related information. These modalities complement patient-entered information collected through the mHealth application and enable the platform to capture heterogeneous aspects of the patient’s health and lifestyle context.

The accelerometer provides activity-related information that contributes to the Exercise Module, while camera-based inputs support the nutrition and diet workflow, including food classification and nutrient estimation. The microphone provides an additional channel for patient interaction, and smartwatch-derived information complements smartphone-based sensing with connected wearable measurements. These heterogeneous inputs are acquired through the mobile application and transferred to the AIDCare backend for subsequent processing, health analysis, personalization, and clinician review.

To aggregate heterogeneous temporal streams into a unified patient state representation at discrete time step *t*, the multimodal sensor vectors from smartphone and wearable devices are fused as

(1) $$X_{t}={\left[x_{acc,t}^{T}\;,\;x_{cam,t}^{T}\;,\;x_{mic,t}^{T}\;,\;x_{sw,t}^{T}\right]}^{T}$$

where $X_{t}$ denotes the composite multimodal sensor observation vector acquired for the patient at time step *t*, $x_{acc,t}$ represents the sub-vector of tri-axial acceleration features extracted from the smartphone accelerometer, $x_{cam,t}$ denotes the visual feature vector derived from smartphone camera captures, $x_{mic,t}$ represents the acoustic feature vector captured via the smartphone microphone, $x_{sw,t}$ symbolizes the physiological and motion feature vector received from the connected smartwatch, ${\left(\cdot \right)}^{T}$ represents the matrix transpose operator performing spatial vector concatenation, and *t* denotes the discrete temporal sampling index.

The integration of smartphone and wearable sensing allows AIDCare to combine physiological, activity-related, visual, and interaction-related information within a common patient context rather than treating each sensing modality as an independent data source. In the reference implementation, hardware devices comprised consumer smartphones (Samsung Galaxy A73 running Android 16; Apple iPhone 17 Pro Max running iOS 27) and commercial smartwatches (Samsung Galaxy Watch 5 OS 6 and Apple Watch Series 8 running watchOS 9.5). Device pairing and telemetry transmission operated via Bluetooth Low Energy (BLE 5.0) using custom Generic Attribute Profile (GATT) services for heart rate and motion streaming, alongside HTTPS/REST endpoints over Wi-Fi and cellular networks. Sampling frequencies were set to 50 Hz for the tri-axial accelerometer, 16 kHz for the microphone during voice interactions, 1 Hz for smartwatch photoplethysmography (PPG) pulse rate, and native 12 MP captures downscaled to 640×640 pixels for camera-based food imaging.

The implemented sensing pipeline was evaluated across 400 controlled bench experimental trials (100 independent trials per modality: smartphone accelerometer, camera, microphone, and smartwatch). These trials evaluated pipeline completion under simulated network drops, background task scheduling, and payload variations. Crucially, these measurements evaluate software data-pipeline completion rates (successful hardware I/O capture, network transmission, and backend database parsing) under controlled engineering conditions. They do not evaluate sensor measurement accuracy, physiological precision, or clinical sensitivity/specificity; no validation against certified reference instruments (such as a laboratory-calibrated YSI glucose analyzer or a clinical continuous glucose monitor [CGM]) was conducted in this study.

**Figure 2:**
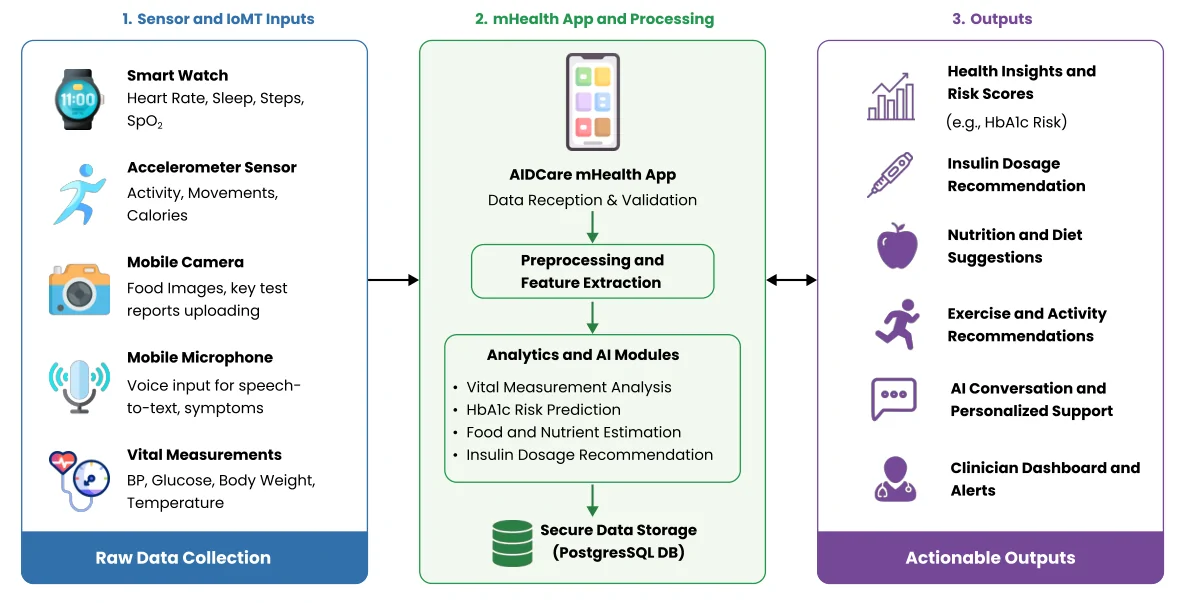
Multimodal smartphone and IoMT sensing pathway for the AIDCare mHealth application.

As illustrated in Figure 2, the sensing layer is connected to the broader AIDCare architecture, allowing information acquired from smartphones and connected wearable devices to contribute to downstream health-analysis and personalized health-management components.

### 3.3. mHealth Data Acquisition and Backend Processing

The AIDCare mHealth application acts as the interface between the patient, sensing devices, and backend services. Information acquired through smartphone sensors, connected wearable devices, and patient-generated inputs is transferred to the backend for subsequent processing. The backend provides a centralized environment for managing patient information, coordinating application functionality, and making relevant information available to downstream analytical and intelligent components.

The backend separates the acquisition of heterogeneous information from its subsequent analysis. This separation allows the same patient context to be accessed by different specialized modules without requiring each sensing modality to implement independent health-management logic. The architecture consequently establishes a common processing pathway through which physiological, activity-related, dietary, and interaction information can be managed within the same patient context.

Structured patient and application information is maintained in the PostgreSQL database. Persistent storage supports the organization of longitudinal patient information and provides the analytical components with access to previously recorded measurements and relevant patient-generated information. This longitudinal representation is important for personalized health management because individual recommendations and interactions may depend not only on the most recent observation but also on previously recorded information and patient-specific goals.

### 3.4. Vital Measurement Processing and Health Data Management

The Vital Measurements Module processes health measurements recorded through the AIDCare application. The module receives patient-recorded measurements and applies a rule-based processing mechanism to support the systematic handling of daily vital measurements. The processed information is subsequently maintained within the backend and database infrastructure for use by other components of the architecture.

To reduce high-frequency sampling noise and fingerstick measurement artifacts in longitudinal health streams, recorded capillary blood glucose measurements are dynamically smoothed using an exponentially weighted moving average (EWMA) temporal filter:

(2) $${\hat{G}}_{t}=\alpha G_{t}+\left(1-\alpha \right){\hat{G}}_{t-1}$$

where ${\hat{G}}_{t}$ represents the filtered blood glucose estimate for the current measurement event *t*, $G_{t}$ denotes the raw capillary glucose measurement recorded at event *t* (in mg/dL), ${\hat{G}}_{t-1}$ is the smoothed glucose baseline estimate from the preceding event t−1, and α denotes the exponential smoothing coefficient, configured to α=0.35. This coefficient value was empirically selected to balance responsiveness to acute postprandial glycemic excursions against attenuation of spurious transient measurement noise. In the reference implementation, measurements reflect a standard self-monitoring schedule (fasting, pre-prandial, 2-h postprandial, and bedtime; 4–6 entries per day). Missing entries within a 12-h window are handled via a zero-order hold (last observation carried forward); if the sampling interval exceeds 12 h, the filter baseline resets to the next raw observation (${\hat{G}}_{t-1}\leftarrow G_{t}$) to prevent stale historical readings from biasing subsequent estimates.

The purpose of this layer is to transform individual measurements into structured patient information that can be consumed by downstream modules. By maintaining the measurements within a longitudinal patient record, AIDCare provides a common information source for HbA1c risk assessment, insulin dosage calculation, personalized recommendation, and clinician review.

The rule-based processing implemented within this component should be distinguished from the broader personalization functionality. Its primary role is to support the systematic processing of daily vital measurements, whereas the AI Personalized Recommender and Medical LLM operate on the broader patient context generated across the architecture.

### 3.5. Multimodal Health Analysis

Following data acquisition and organization, the AIDCare architecture applies several specialized health-analysis modules. These modules process different aspects of the patient’s physiological, nutritional, and activity-related information. The purpose of this stage is to transform heterogeneous patient information into structured analytical outputs that can subsequently contribute to personalized health-management support.

#### 3.5.1. Illustrative HbA1c Risk Prior

The HbA1c Risk Module computes an illustrative risk score from patient vital logs to demonstrate the data-flow pathway between physiological monitoring and clinical alerting. The probability of an elevated glycated hemoglobin level ($R_{\mathrm{HbA}1c}=1$, clinically defined as HbA1c≥7.0% [53mmol/mol] per American Diabetes Association guidelines [3]) given the longitudinal physiological feature vector $z_{t}$ is formulated as an illustrative logistic prior:

(3) $$P\left(R_{\mathrm{HbA}1c}=1|z_{t}\right)=\frac{1}{1+e^{-(\beta_{0}+\beta_{1}\bar{G}+\beta_{2}\sigma_{G}+\beta_{3}\mathrm{BMI})}}$$

where $P(R_{\mathrm{HbA}1c}=1|z_{t})$ denotes the conditional probability of exceeding the 7.0% HbA1c threshold given the feature vector $z_{t}={\left[\bar{G},\sigma_{G},\mathrm{BMI}\right]}^{T}$, *e* represents Euler’s constant, $\bar{G}$ represents the mean blood glucose concentration in mg/dL evaluated over a 14-day rolling window, $\sigma_{G}$ denotes the standard deviation of blood glucose readings in mg/dL over the same 14-day window (quantifying short-term glycemic variability), and BMI denotes Body Mass Index in ${\mathrm{kg}/m}^{2}$, populated from patient baseline anthropometric records entered during mobile onboarding and periodically updated.

The numerical coefficients were selected with reference to the physiological relationships established in the landmark A1c-Derived Average Glucose (ADAG) study by the authors of [52] and American Diabetes Association clinical guidelines [3]. Specifically, the validated linear equation linking estimated average glucose to glycated hemoglobin (eAG=28.7×HbA1c−46.7) establishes that an average blood glucose of $\bar{G}\approx 154\;\mathrm{mg}/\mathrm{dL}$ corresponds directly to the established clinical risk threshold of HbA1c=7.0% (53mmol/mol). To translate this linear reference into a smooth probabilistic alerting prior, illustrative logistic coefficients were parameterized using published physiological relationships and cohort summary characteristics from the ADAG study [52]: intercept $\beta_{0}=-8.5$, $\beta_{1}=0.045\;{\left(\mathrm{mg}/\mathrm{dL}\right)}^{-1}$, $\beta_{2}=0.032\;{\left(\mathrm{mg}/\mathrm{dL}\right)}^{-1}$, and $\beta_{3}=0.055{(\mathrm{kg}/m}^{2}{)}^{-1}$. These coefficients were not estimated from individual-level ADAG data and are used only as illustrative parameters for demonstrating the data-flow and clinician alert-triggering mechanism. A 14-day rolling window was chosen in accordance with international consensus on continuous and capillary glucose monitoring [3], which demonstrates that 14 days of glucose sampling provides a stable surrogate for quarterly glycated hemoglobin trends while remaining responsive to recent lifestyle changes.

Here, “parameterized using ADAG reference data” refers only to the numerical parameterization of the illustrative logistic prior using published physiological relationships and cohort summary characteristics from the ADAG study. It does not imply clinical calibration or external validation against patient-level HbA1c outcomes. Accordingly, the formulation is termed an “uncalibrated illustrative prior” because its clinical discrimination, calibration, and predictive validity have not been established against an independent clinical cohort or laboratory-measured HbA1c outcomes.

Crucially, Equation (3) must be understood as an uncalibrated illustrative prior implemented to evaluate data-flow pipelining and clinician alert triggering rather than as an empirical, calibrated prediction model. While mean blood glucose is physiologically linked to estimated average glucose (eAG) via the established ADAG regression [52], incorporating glycemic variability ($\sigma_{G}$) and BMI in an unvalidated logit does not constitute an externally validated clinical prognostic tool. Discrimination metrics (e.g., AUC-ROC/AUPRC) and Brier calibration against certified venous laboratory HbA1c assays have not been established and represent a primary objective of Phase 4 in our translational roadmap.

#### 3.5.2. Carbohydrate-Based Prandial Insulin Bolus Calculator

The Insulin Bolus Calculator provides computational dosage estimates by linking meal carbohydrate quantification with physiological blood glucose telemetry. To ensure patient safety and avoid overstating algorithmic autonomy, this module is implemented strictly as a clinician-supervised calculator rather than an autonomous prescription engine.

The recommended prandial bolus insulin dosage $D_{\mathrm{ins}}$ is computed by combining meal carbohydrate coverage with corrective glucose adjustment, bounded by active insulin-on-board and safety guardrails:

(4) $$D_{\mathrm{ins}}=\min\left(D_{\max}\;,\;\max\left(0\;,\;\frac{{\mathrm{CHO}}_{\mathrm{total}}}{\mathrm{ICR}}+\frac{{\hat{G}}_{t}-G_{\mathrm{target}}}{\mathrm{ISF}}-{\mathrm{IOB}}_{t}\right)\right)$$

where $D_{\mathrm{ins}}$ denotes the calculated prandial insulin bolus in Units (U), ${\mathrm{CHO}}_{\mathrm{total}}$ represents the total meal carbohydrate mass in grams (g) estimated via the nutrition vision pipeline and confirmed by the patient, ${\hat{G}}_{t}$ is the current smoothed blood glucose level in mg/dL, and $G_{\mathrm{target}}$ is the target glucose baseline (e.g., 100–120 mg/dL). The patient-specific clinical parameters comprise the Insulin-to-Carbohydrate Ratio (ICR, in g/U) and the Insulin Sensitivity Factor (ISF, in mg/dL/U), both configured and locked by attending healthcare providers via the Clinician Dashboard. The term ${\mathrm{IOB}}_{t}$ denotes active Insulin-on-Board in Units, modeled as an exponential decay over a 3-to-4-h pharmacokinetic duration to prevent insulin stacking from prior boluses.

Rigid clinical safety guardrails are architecturally embedded into the calculator:

1. **Hypoglycemia Lockout:** If current smoothed glucose ${\hat{G}}_{t}\leq 70\;\mathrm{mg}/\mathrm{dL}$ (or downward trend exceeds 2 mg/dL/min), the bolus calculation is unconditionally locked out, and the UI immediately issues an alert prompting ingestion of 15–20 g of rapid-acting carbohydrates following the clinical “Rule of 15”.
2. **Maximum Bolus Ceiling:** A clinician-preset maximum single bolus cap $D_{\max}$ (defaulting to 15 U) prevents catastrophic overdosing due to erroneous carbohydrate inputs or system faults.
3. **Propagation of Carbohydrate Uncertainty:** Because computer-vision carbohydrate estimation exhibits known variance (14–25% MAPE [9,10]), the mobile application displays the estimated carbohydrate mass alongside an interactive adjustment slider, requiring manual patient confirmation before dosage calculation.

This component operates as an educational and computational aid under strict clinician oversight and has not yet been validated against clinical dosing records or regulatory-grade metabolic simulators (e.g., the UVA/Padova type 1 diabetes simulator).

#### 3.5.3. Nutrition and Diet Assessment

The Nutrition and Diet Module serves as a cornerstone of the AIDCare platform, translating raw meal captures into structured nutritional telemetry. In diabetes management, nutritional self-care extends beyond rudimentary calorie counting: the specific composition of macronutrients—particularly carbohydrates, dietary fiber, and protein—governs postprandial glucose excursions, insulin requirements, and long-term metabolic health. The module adapts and extends the vision-based dietary estimation architectures from our previous foundational work [9,10] to achieve multi-nutrient quantification from smartphone camera imagery.

When a patient captures a meal image through the AIDCare mobile application, a computer-vision pipeline performs multi-class food detection, semantic segmentation, and bounding contour extraction for each visible food item k∈{1,2,…,K}. The 3D spatial volume $V_{k}$ (in ${\mathrm{cm}}^{3}$) of each segmented food item is estimated using geometric shape modeling and camera-to-plate projection priors. The mass $m_{k}$ (in grams) of item *k* is derived from its estimated volume and food-specific physical mass density $\rho_{k}$ (${g/\mathrm{cm}}^{3}$) indexed from standardized food density tables. Subsequently, nutritional composition vectors are retrieved by mapping the identified food class to standardized reference food composition databases, specifically USDA FoodData Central and the Turkish National Food Composition Database (TÜRKOMP).

To quantify the complete macronutrient profile relevant to glycemic control, the module computes the total meal mass for carbohydrates (${\mathrm{CHO}}_{\mathrm{total}}$), dietary protein (${\mathrm{PRO}}_{\mathrm{total}}$), and dietary fiber (${\mathrm{FIB}}_{\mathrm{total}}$) across all *K* identified food items:

(5) $${\mathrm{CHO}}_{\mathrm{total}}=\sum_{k=1}^{K}V_{k}\cdot \rho_{k}\cdot c_{k,\mathrm{cho}}$$

(6) $${\mathrm{PRO}}_{\mathrm{total}}=\sum_{k=1}^{K}V_{k}\cdot \rho_{k}\cdot c_{k,\mathrm{pro}}$$

(7) $${\mathrm{FIB}}_{\mathrm{total}}=\sum_{k=1}^{K}V_{k}\cdot \rho_{k}\cdot c_{k,\mathrm{fib}}$$

where $V_{k}$ denotes the estimated 3D spatial volume of food item *k* in ${\mathrm{cm}}^{3}$, $\rho_{k}$ represents the mass density in ${g/\mathrm{cm}}^{3}$, and $c_{k,\mathrm{cho}}$, $c_{k,\mathrm{pro}}$, and $c_{k,\mathrm{fib}}$ represent the fractional content ratios per unit mass for carbohydrate, protein, and dietary fiber, respectively.

Each calculated nutrient serves a distinct physiological and clinical role within the platform’s decision-support pathway:

- **Carbohydrates (${\mathrm{CHO}}_{\mathrm{total}}$):** Represent the primary source of exogenous postprandial glucose rise, directly serving as the input for the prandial bolus insulin calculator in Equation (4).
- **Dietary Fiber (${\mathrm{FIB}}_{\mathrm{total}}$):** Plays a critical role in blunting acute postprandial glycemic spikes by retarding gastric emptying and glucose absorption. It enables the calculation of net available carbohydrates (${\mathrm{CHO}}_{\mathrm{net}}={\mathrm{CHO}}_{\mathrm{total}}-{\mathrm{FIB}}_{\mathrm{total}}$), which clinicians can configure for patients requiring refined bolus calculations.
- **Dietary Protein (${\mathrm{PRO}}_{\mathrm{total}}$):** Promotes satiety, supports lean muscle preservation during weight management, and induces late-phase gluconeogenesis, which informs contextual lifestyle advice and prevents nocturnal hypoglycemia.

These structured nutritional outputs are logged in the longitudinal patient database, evaluated against individualized dietary targets via the nutritional compliance score $S_{\mathrm{nutr}}\left(a,{\mathrm{CHO}}_{\mathrm{total}}\right)$ in Equation (10), and made accessible to attending healthcare professionals through the Clinician Dashboard.

#### 3.5.4. Physical Activity and Energy Expenditure Assessment

The Exercise Module processes activity-related information acquired through the sensing layer. Information from the smartphone accelerometer and connected smartwatch can contribute to the representation of the patient’s physical activity. The resulting activity information complements nutrition and physiological measurements and provides a broader view of lifestyle-related factors relevant to diabetes self-management.

To translate raw wearable motion signals into active energy expenditure (${\mathrm{EE}}_{\mathrm{act}}$), tri-axial acceleration vectors $x_{\mathrm{acc},t}={\left[a_{x,t},a_{y,t},a_{z,t}\right]}^{T}$ (sampled at 50 Hz) are filtered with a 4th-order Butterworth bandpass filter (0.25–11 Hz) to remove gravitational and high-frequency noise. The dynamic acceleration vector magnitude is computed as $a_{\mathrm{vm},t}=\left|\sqrt{a_{x,t}^{2}+a_{y,t}^{2}+a_{z,t}^{2}}-1g\right|$, integrated over non-overlapping 10-s epochs.

Epoch vector magnitudes are mapped to discrete Metabolic Equivalent of Task (MET) tiers using operational accelerometer cut-points implemented for the AIDCare software pipeline. These thresholds are intended for functional activity classification and have not been independently validated in the present study:

- Sedentary Behavior ($a_{\mathrm{vm}}<0.05g$): 1.2 METs (resting, sitting).
- Light Physical Activity ($0.05g\leq a_{\mathrm{vm}}<0.25g$): 2.2 METs (slow walking, household tasks).
- Moderate Physical Activity ($0.25g\leq a_{\mathrm{vm}}<0.75g$): 4.0 METs (brisk walking, casual cycling).
- Vigorous Physical Activity ($a_{\mathrm{vm}}\geq 0.75g$): 7.0 METs (running, vigorous aerobics).

Active energy expenditure during an exercise session of duration Δτ (in minutes) is then calculated as:

(8) $${\mathrm{EE}}_{\mathrm{act}}=\mathrm{MET}\left(x_{\mathrm{acc},t}\right)\times 3.5\times \frac{W}{200}\times \Delta \tau$$

where ${\mathrm{EE}}_{\mathrm{act}}$ is expressed in kilocalories (kcal), 3.5 represents resting oxygen uptake baseline in $\mathrm{mL}\;O_{2}\cdot {\mathrm{kg}}^{-1}\cdot \min^{-1}$, *W* denotes patient weight in kilograms, and 200 is the metabolic conversion factor ($\mathrm{kcal}\cdot \min^{-1}$ per $\mathrm{mL}\;O_{2}\cdot {\mathrm{kg}}^{-1}\cdot \min^{-1}$). It must be emphasized that Equation (8) provides an operational approximation within the mHealth software pipeline to guide lifestyle scoring; it does not replace research-grade indirect calorimetry or doubly labelled water reference standards.

### 3.6. H5P Content Parsing and Quantized Low-Rank Adaptation

The AIDCare platform incorporates an H5P Parsing Engine to support the integration of structured medical content. H5P files can be uploaded to the backend, where the parsing engine extracts relevant content and converts the information into a representation that can be processed by the application.

The training corpus was derived from the open-access Turkish Hospital Medical Articles Dataset [42] (Hugging Face identifier: alibayram/turkish-hospital-medical-articles), containing 24,612 Turkish-language educational and clinical articles compiled across verified hospital networks for patient lifestyle education (not Electronic Health Records [EHR] or private patient data). A domain audit identified that approximately 6420 articles specifically address diabetes pathophysiology, carbohydrate counting, medical nutrition therapy, insulin regimens, and glycemic targets, while the remainder encompass related metabolic, cardiovascular, and preventive health domains. Because raw H5P educational packages contain nested JSON schemas and repetitive boilerplate, Second Normal Form (2NF) relational normalization decomposes hierarchical packages into standardized instructional pairs (clinical context → tailored recommendation), removing redundancy while preserving medical meaning.

The 24,612 articles were partitioned at the document level (ensuring zero data leakage across splits) using a fixed random seed of 42 into: 80% training (*n* = 19,690), 10% validation (*n* = 2461), and 10% test (*n* = 2461). The final test set was strictly held out and sequestered during model development. Domain adaptation was performed using Quantized Low-Rank Adaptation (QLoRA) on the MedGemma 4B-IT base model, minimizing the causal language modeling objective:

(9) $$L_{\mathrm{QLoRA}}\left(\theta ,A,B\right)=-\sum_{j=1}^{N}\log P\left(y_{j}|y_{<j},x\;;\;\theta +AB\right)$$

where θ denotes the frozen base weights quantized to 4-bit NormalFloat (NF4) with double quantization, $A\in R^{d\times r}$ and $B\in R^{r\times k}$ are low-rank adapter matrices with rank r=16 and scaling $\alpha_{\mathrm{LoRA}}=32$ (scaling factor α/r=2.0), applied to all linear attention and feed-forward projection layers (q_proj, k_proj, v_proj, o_proj, gate_proj, up_proj, down_proj) with a dropout of 0.05. Training used the 8-bit AdamW optimizer ($\beta_{1}=0.9,\beta_{2}=0.999$, $\epsilon =10^{-8}$, weight decay 0.01), an initial learning rate of $2\times 10^{-4}$ with a cosine decay schedule and 100 warmup steps, per-device batch size of 4 with gradient accumulation of 4 (yielding an effective batch size of 16), and a maximum sequence length of 1024 tokens. Training ran for 20 epochs with random seed 42 on a dedicated workstation equipped with an NVIDIA RTX 5000 GPU (64 GB GDDR6 VRAM) running PyTorch 2.2 and Hugging Face PEFT 0.20.

Inference prompts adhere to a structured clinical instruction format that incorporates patient context directly into the prompt without requiring external vector database retrieval, as illustrated in Listing 1.

**Listing 1.** Structured clinical instruction prompt used in AIDC are inference.
<start_of_turn>user

[AIDCare Patient Context]

Vitals: Glucose = {Ghat_t} mg/dL, Mean-14d = {Gbar} mg/dL,

BMI = {BMI} kg/m^2.

Diet: Carbs = {CHO} g, Fiber = {FIB} g, Protein = {PRO} g.

Activity: {EE_act} kcal. Patient Query: {query}

Task: Classify intent into [Diet, Activity, Monitoring, Consult]

and generate clinician-supervised lifestyle response.

<end_of_turn>

<start_of_turn>model

[Intent: {Intent_Class}] {Clinical_Guidance}

<end_of_turn>


Domain adaptation was performed using the Turkish-language medical corpus, and the quantitative evaluation was conducted on the corresponding document-level benchmark within the same evaluation setting. The English-language inference template shown above was used as an interface-format prompt and does not constitute evidence of cross-lingual clinical guidance. No separate cross-lingual clinical evaluation was performed in the present study.

### 3.7. Medical LLM-Based Patient Interaction and Personalization

A fine-tuned Medical Large Language Model is integrated into AIDCare to provide contextualized interaction with patients. The Medical LLM receives relevant patient information through the AIDCare backend and can utilize patient conversation history to maintain contextual continuity across interactions.

Patient conversation history is maintained as part of the broader patient context. This allows subsequent interactions to consider information from previous conversations rather than treating every interaction as an isolated request. The Medical LLM can therefore support personalized interaction based on the information available within the patient’s digital health context.

The Medical LLM is not designed as an independent clinical decision-maker. Instead, it operates as one component within the integrated architecture and can access information generated by the sensing, health-analysis, and personalization layers. Its outputs are consequently situated within a system that also incorporates structured health information, patient-specific goals, and clinician oversight.

To select candidate lifestyle interventions a∈A within the mobile interface, the AI Personalized Recommender evaluates a multi-objective utility scoring function:

(10) $$S_{\mathrm{rec}}\left(a\right)=w_{1}S_{\mathrm{vital}}\left(a,{\hat{G}}_{t}\right)+w_{2}S_{\mathrm{nutr}}\left(a,{\mathrm{CHO}}_{\mathrm{total}}\right)+w_{3}S_{\mathrm{act}}\left(a,{\mathrm{EE}}_{\mathrm{act}}\right)+w_{4}S_{\mathrm{pref}}\left(a\right)$$

The candidate action catalog A comprises 60 parameterized clinical lifestyle recommendations categorized into four distinct self-management sub-domains: (i) dietary adjustments $A_{\mathrm{diet}}$ (20 actions, e.g., low-glycemic carbohydrate substitutions, portion moderation, pre-meal water hydration, and soluble fiber additions); (ii) physical activity prompts $A_{\mathrm{act}}$ (15 actions, e.g., 15-min postprandial walking, light resistance band routines, and aerobic cycling); (iii) glycemic monitoring actions $A_{\mathrm{mon}}$ (15 actions, e.g., pre-bedtime capillary glucose checks, 2-h postprandial verification, and ketone checks during elevated readings); and (iv) clinician consultation alerts $A_{\mathrm{clin}}$ (10 actions, e.g., prompt to contact attending physician for recurrent hypoglycemia or request insulin sensitivity recalibration).

The four component sub-utilities are operationally formulated on the unit interval [0,1] as follows:

1. **Physiological Safety Score $S_{\mathrm{vital}}\left(a,{\hat{G}}_{t}\right)$:** Evaluates candidate action safety against real-time smoothed capillary glucose ${\hat{G}}_{t}$. For dietary actions $a\in A_{\mathrm{diet}}$, $S_{\mathrm{vital}}=1.0$ when ${\hat{G}}_{t}\in \left[70,140\right]\;\mathrm{mg}/\mathrm{dL}$, decreasing linearly to 0.0 at ${\hat{G}}_{t}\geq 250\;\mathrm{mg}/\mathrm{dL}$; if ${\hat{G}}_{t}<70\;\mathrm{mg}/\mathrm{dL}$, $S_{\mathrm{vital}}=0.0$ unless *a* represents a fast-acting rescue carbohydrate intervention ($S_{\mathrm{vital}}=1.0$). For physical activity actions $a\in A_{\mathrm{act}}$, $S_{\mathrm{vital}}=1.0$ within the safe exercise window [100,180]mg/dL, dropping to 0.0 if ${\hat{G}}_{t}<90\;\mathrm{mg}/\mathrm{dL}$ (hypoglycemia risk) or ${\hat{G}}_{t}>250\;\mathrm{mg}/\mathrm{dL}$ (ketoacidosis risk).
2. **Nutritional Compliance Score $S_{\mathrm{nutr}}\left(a,{\mathrm{CHO}}_{\mathrm{total}}\right)$:** Assesses alignment with clinician-prescribed daily carbohydrate budgets (${\mathrm{CHO}}_{\mathrm{target}}$) and dietary fiber goals:
  (11) $$S_{\mathrm{nutr}}\left(a\right)=\max\left(0\;,\;1-\frac{|{\mathrm{CHO}}_{\mathrm{cum}}+{\mathrm{CHO}}_{a}-{\mathrm{CHO}}_{\mathrm{target}}|}{{\mathrm{CHO}}_{\mathrm{target}}}\right)\cdot \min\left(1.0\;,\;0.5+0.5\frac{{\mathrm{Fiber}}_{a}}{10\;g}\right)$$
  where ${\mathrm{CHO}}_{\mathrm{cum}}$ is cumulative carbohydrate intake logged today, ${\mathrm{CHO}}_{a}$ is the carbohydrate content of action *a*, and ${\mathrm{Fiber}}_{a}$ is its dietary fiber content.
3. **Physical Activity Score $S_{\mathrm{act}}\left(a,{\mathrm{EE}}_{\mathrm{act}}\right)$:** Measures progress toward clinician-prescribed daily active energy expenditure targets (${\mathrm{EE}}_{\mathrm{target}}$ in kcal):
  (12) $$S_{\mathrm{act}}\left(a\right)=\min\left(1.0\;,\;\frac{{\mathrm{EE}}_{\mathrm{cum}}+{\mathrm{EE}}_{a}}{{\mathrm{EE}}_{\mathrm{target}}}\right)$$
  where ${\mathrm{EE}}_{\mathrm{cum}}$ is the cumulative wearable active energy expenditure logged for the day and ${\mathrm{EE}}_{a}$ is the expected caloric burn of action *a*.
4. **Patient Preference and Adherence Score $S_{\mathrm{pref}}\left(a\right)$:** Models patient historical receptivity using Laplace-smoothed historical acceptance rates:
  (13) $$S_{\mathrm{pref}}\left(a\right)=\frac{N_{\mathrm{accepted}}\left(a\right)+1}{N_{\mathrm{offered}}\left(a\right)+2}$$
  where $N_{\mathrm{offered}}\left(a\right)$ is the total number of times action *a* was recommended to the user, and $N_{\mathrm{accepted}}\left(a\right)$ is the number of times the patient confirmed or completed the action.

Weights $w_{1},w_{2},w_{3},w_{4}\geq 0$ satisfy $\sum_{m=1}^{4}w_{m}=1$, initialized uniformly ($w_{m}=0.25$) and calibrated by the clinician. When a healthcare provider adjusts or rectifies recommendations, weights update via gradient feedback:

(14) $$w^{\left(new\right)}={\mathrm{Proj}}_{\Delta }\left(w^{\left(old\right)}+\gamma \;\delta_{\mathrm{clinician}}\;\nabla_{w}S_{\mathrm{rec}}\left(a^{*}\right)\right)$$

where learning rate γ=0.1, feedback scalar $\delta_{\mathrm{clinician}}\in \left\{+1,-1\right\}$ (+1 for clinician approval, −1 for clinician rejection or override), and ${\mathrm{Proj}}_{\Delta }$ performs projection onto the unit simplex $\Delta^{3}$: non-negativity truncation $w_{m}\leftarrow \max\left(0,w_{m}\right)$ followed by $L_{1}$ re-normalization $w_{m}\leftarrow w_{m}/\sum_{j=1}^{4}w_{j}$, strictly preserving $\sum_{m=1}^{4}w_{m}=1$ and $w_{m}\geq 0$. This scoring model operates as an architectural framework; offline and online ranking metrics (e.g., nDCG, pairwise clinician preference) are slated for prospective evaluation in Phase 3.

The clinician-in-the-loop mechanism is particularly important for a system that combines heterogeneous sensor information with AI-generated outputs. Sensor-derived information may be incomplete or context-dependent, while AI-generated responses may require verification. Incorporating clinician oversight therefore provides an additional control layer between automated system outputs and health-management decisions.

## 4. Implementation

The AIDCare platform was implemented as an integrated mobile health system connecting the patient-facing mHealth client, smartphone and IoMT sensing components, backend analytical services, health-analysis modules, intelligent personalization components, LLM-based assistance, and clinician-in-the-loop functionality. The mobile client was developed using Flutter (v3.47, Dart SDK v3.13) to ensure uniform deployment across Android and iOS. Communication between the mobile application and backend was implemented through RESTful HTTPS APIs with structured JSON payloads, developed using ASP.NET Core 10.0 and Bootstrap 5. Persistent storage and longitudinal health-data schemas were maintained using PostgreSQL 18.6. The conversational intelligence and intent-classification service was implemented using Python 3.10, PyTorch 2.2.0, Hugging Face Transformers 4.38.1, PEFT 0.20, and BitsAndBytes 0.50.0.

The implementation was organized according to the functional components of the proposed architecture. These components include the mobile application interface, smartphone and IoMT sensing layer, health monitoring and analysis modules, Medical LLM and personalization services, medical content infrastructure, and clinician dashboard. This organization enables the implemented system to maintain a common patient context across data acquisition, health analysis, personalized interaction, and clinician oversight.

The AIDCare platform implements interconnected health-monitoring and analysis components including Medical LLM Assistant, the Vital Measurements Module, HbA1c Risk Prior Module, Carbohydrate-Based Insulin Bolus Calculator, Nutrition and Diet Module, and Exercise Module. These components process physiological, dietary, and activity-related information acquired through the AIDCare application and sensing layer and provide structured outputs for subsequent personalization and clinician review.

### 4.1. AIDCare Platform Implementation

The AIDCare mobile application was developed using Flutter to provide a unified interface for patient-facing diabetes self-management functionality. The application provides interfaces through which patients can record and access health information, interact with the sensing components, review personalized information, access conversational AI facilities through a fine-tuned Medical LLM, manage their lifestyle using the diet and exercise modules, and receive HbA1c and insulin dosage recommendations based on vital measurements. The mobile application also serves as the primary gateway through which smartphone-generated information and connected device information enter the AIDCare backend.

The application communicates with the backend through RESTful APIs implemented using the .NET framework. This architecture separates the presentation layer from backend processing and data management while allowing the mobile application to exchange patient information with the relevant services. The resulting implementation provides a common mobile interface for the different health-management components rather than requiring patients to use separate applications for healthcare monitoring, and personalized support.

**Figure 3:**
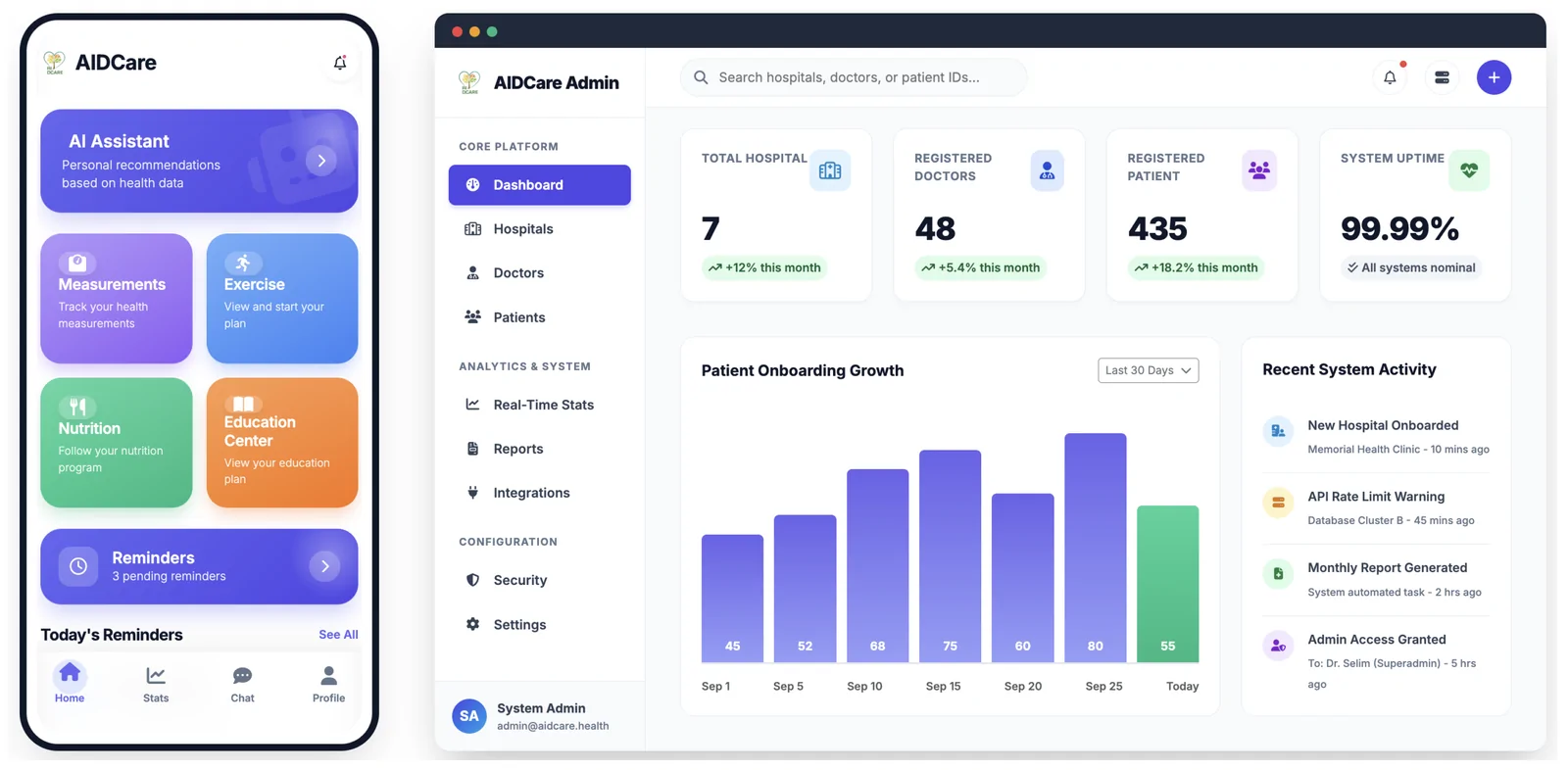
The complete AIDCare platform interface, featuring the patient mobile application alongside the centralized administrative dashboard for real-time healthcare network monitoring.

Figure 3 shows the cross-platform user interfaces of the AIDCare ecosystem, demonstrating the mobile client designed for patient interaction alongside the comprehensive administrative dashboard used to track hospital network statistics and system health. The figure presents representative screens of the AIDCare mobile application and demonstrates the integration of the different patient-facing functions within the mobile environment.

#### 4.1.1. Medical LLM and Personalization Implementation

The intelligent interaction functionality was implemented by interfacing the AIDCare platform with the Hugging Face Transformers library to access Large Language Model functionality. A fine-tuned Medical LLM is integrated into the application to support contextualized interaction based on relevant patient information and conversation history. The model is deployed on the server backend using a dedicated FastAPI service serving the 4-bit quantized QLoRA adapter on the MedGemma 4B-IT base model. System-level response latency was quantitatively benchmarked across N=500 consecutive patient interaction queries transmitted from mobile clients over local Wi-Fi 6 (802.11ax, 5 GHz, RTT <5 ms) and cellular LTE (Band 3/20, RTT 35±12 ms) to the server workstation (NVIDIA RTX 5000 GPU with 64 GB GDDR6, Intel Xeon E5-2680 v4 @ 2.40 GHz, 64 GB RAM). Input prompts averaged 420±45 tokens (incorporating patient physiological telemetry, meal macronutrient estimates, active energy expenditure, and user queries), and generation was capped at a maximum of 256 tokens using greedy decoding (temperature 0.2, top-p 0.95). Across the 500 benchmarked queries, the mean end-to-end response latency (encompassing HTTP round-trip network transit, tokenization, QLoRA autoregressive generation, and JSON response deserialization) was 1.74±0.18 s (range: 1.38–2.14 s; median: 1.71 s; 95th percentile: 1.98 s). This latency represents an engineering responsiveness benchmark under controlled bench conditions rather than an indicator of clinical efficacy or advisory safety. The LLM functionality is connected to the AIDCare backend rather than operating as an independent application component, allowing patient-specific context to be incorporated into the interaction workflow.

The platform maintains patient conversation history to support continuity across interactions. This allows the intelligent assistant to consider relevant information from previous interactions when providing subsequent responses. The Medical LLM is also connected to the broader personalization architecture, allowing information generated by the health-monitoring and analysis modules to contribute to the patient context.

The AI Personalized Recommender provides an additional layer for generating individualized health-management support. The recommender can incorporate information from vital measurements, health-analysis modules, nutrition and exercise information, Medical LLM interactions, and patient-specific lifestyle goals. This implementation establishes a connection between the sensing and analytical layers and the patient-facing personalized support functionality.

**Figure 4:**
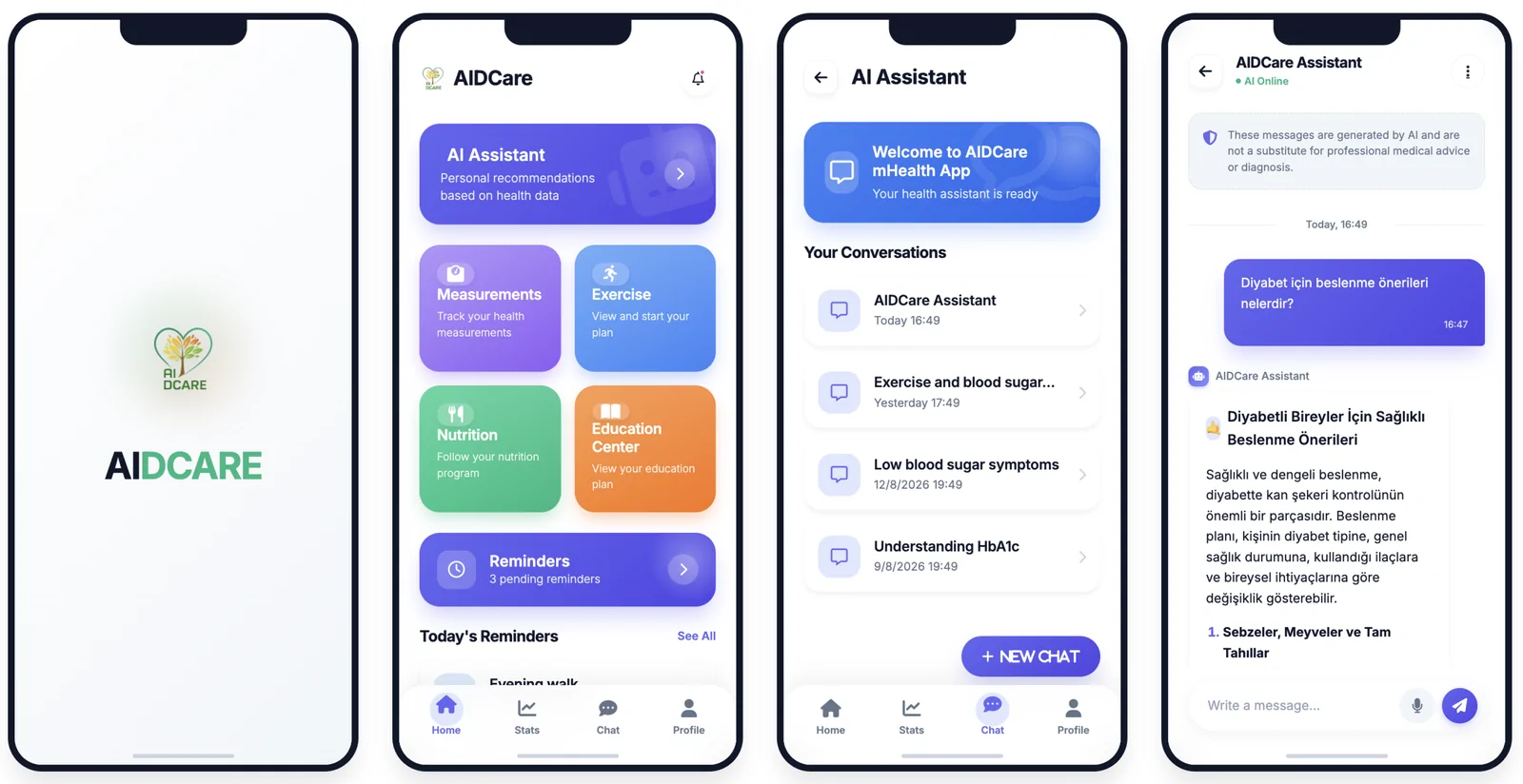
Implementation interface of the Medical LLM and AI Personalized Recommender components of AIDCare, demonstrating the integration of contextual patient information, conversation history, health-monitoring data, nutrition and activity information, and patient-specific goals within the personalized health-management workflow.

Figure 4 demonstrates the implementation of the AI Assistant module, highlighting how the Medical LLM processes contextual information to support personalized health-management interaction.

#### 4.1.2. Vital Measurements Module

**Figure 5:**
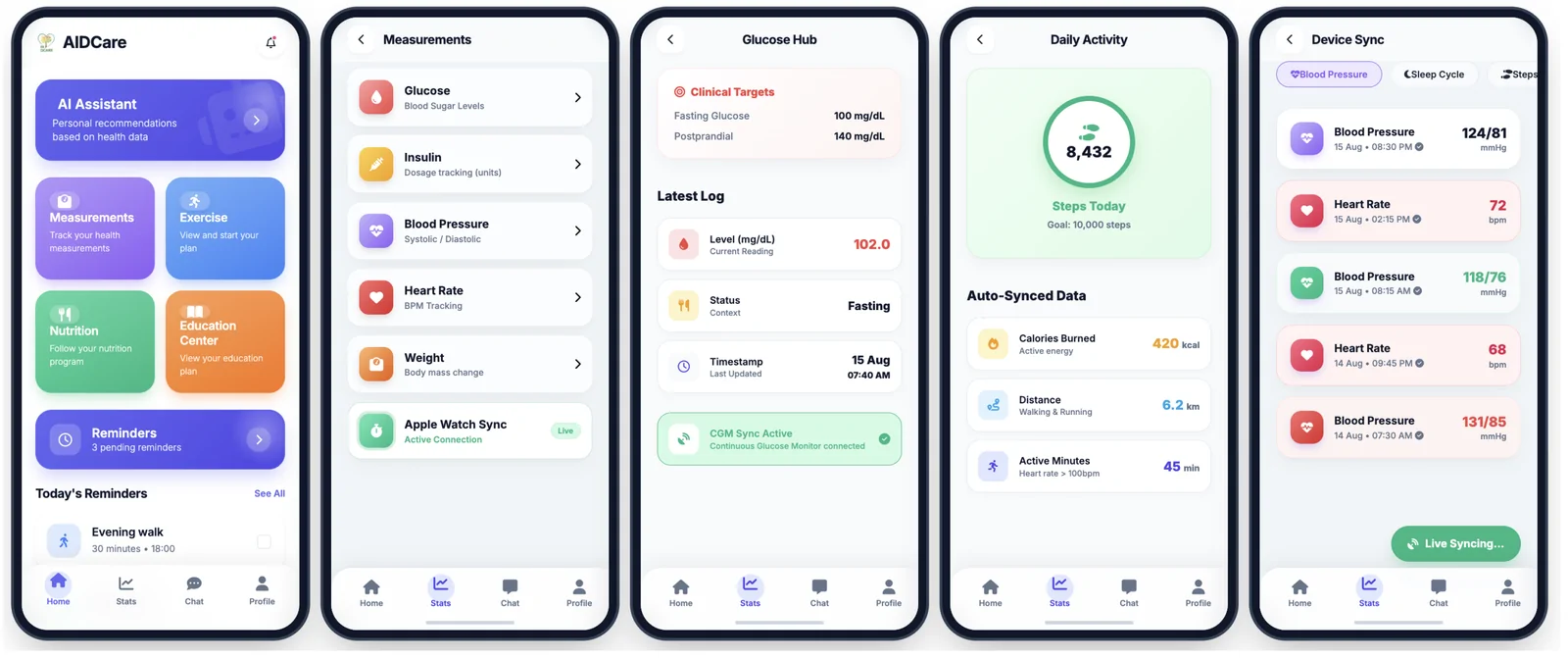
Implementation interface of the Vital Measurements Module, illustrating the recording and presentation of patient health measurements within the AIDCare platform.

The Vital Measurements Module provides the primary interface for recording and managing daily patient health measurements. The recorded measurements are maintained within the patient’s longitudinal health record and are made available to downstream analytical and personalization components. Figure 5 demonstrates the implementation interface of the Vital Measurements Module, detailing how metrics like glucose, insulin, and continuous sensor data are cleanly presented to the user.

#### 4.1.3. HbA1c Risk Prior and Insulin Bolus Calculator Modules

**Figure 6:**
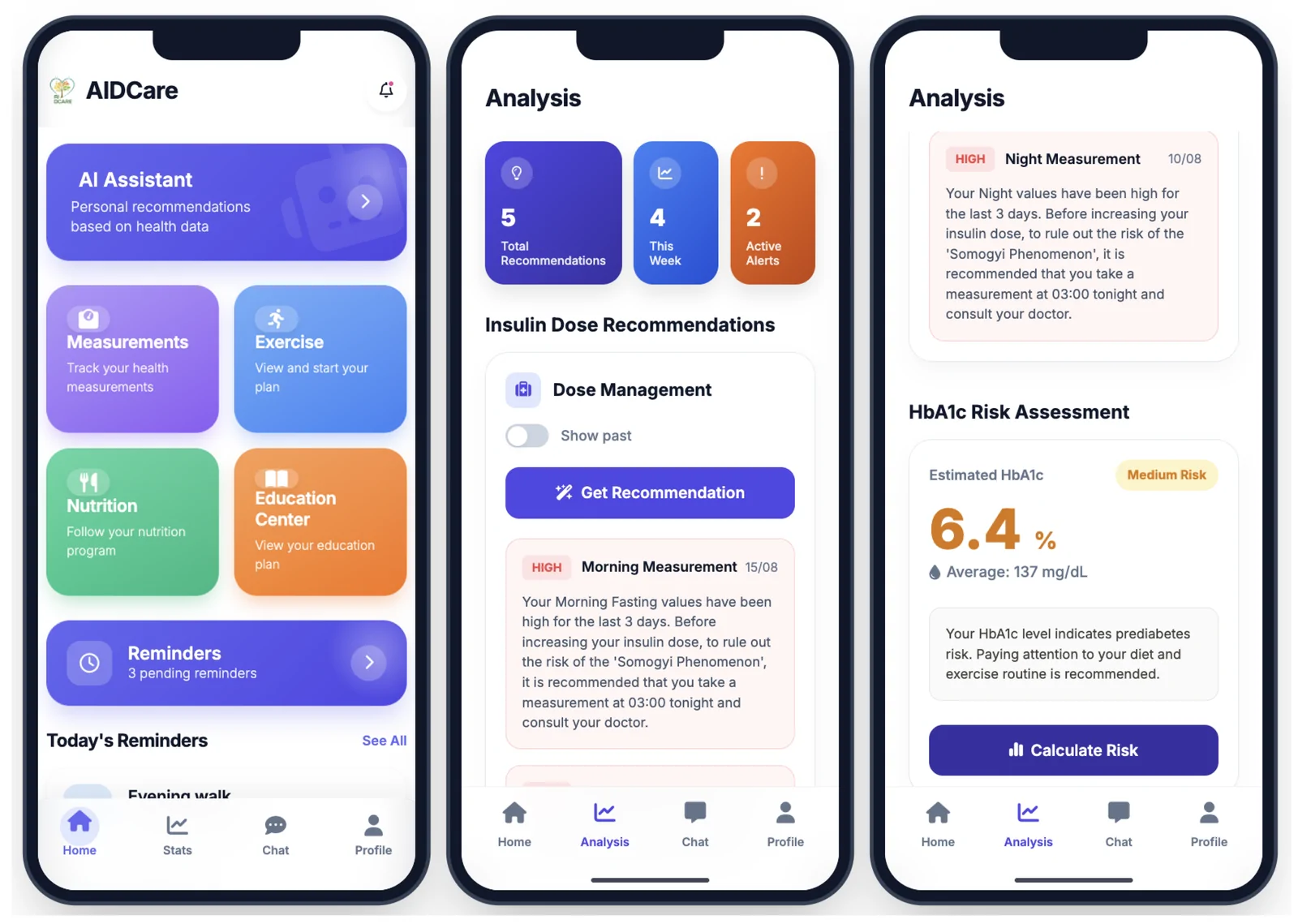
Implementation interface of the HbA1c Risk Prior Module and Carbohydrate-Based Insulin Bolus Calculator within the AIDCare platform.

The HbA1c Risk Module processes patient vital logs to display illustrative risk information, while the Carbohydrate-Based Insulin Bolus Calculator combines estimated carbohydrate content with glucose telemetry to compute prandial insulin suggestions under clinician oversight. As illustrated in Figure 6, the application displays risk alerts and safety warnings within the insulin bolus calculator workflow.

#### 4.1.4. Exercise and Activity Module

**Figure 7:**
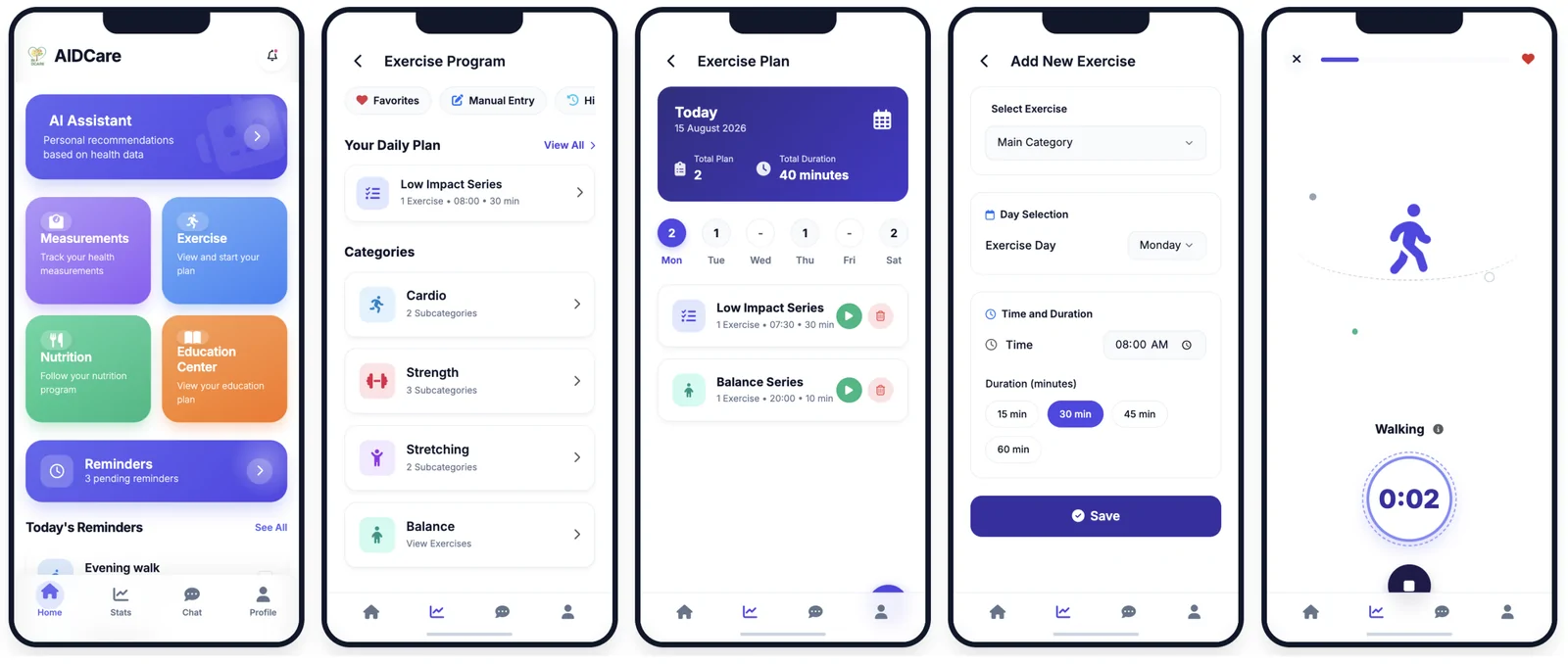
Implementation interface of the Exercise and Activity Module, illustrating the presentation of active-session tracking and historical physical activity data derived from smartphone and smartwatch sensing.

The Exercise Module processes activity-related information obtained from the sensing layer. Smartphone accelerometer and connected smartwatch information can contribute to the representation and assessment of the patient’s physical activity. Figure 7 demonstrates the implementation interface of the Exercise and Activity Module, highlighting how active session tracking and historical activity data are presented within the physical activity management interface.

#### 4.1.5. Nutrition and Diet Module

**Figure 8:**
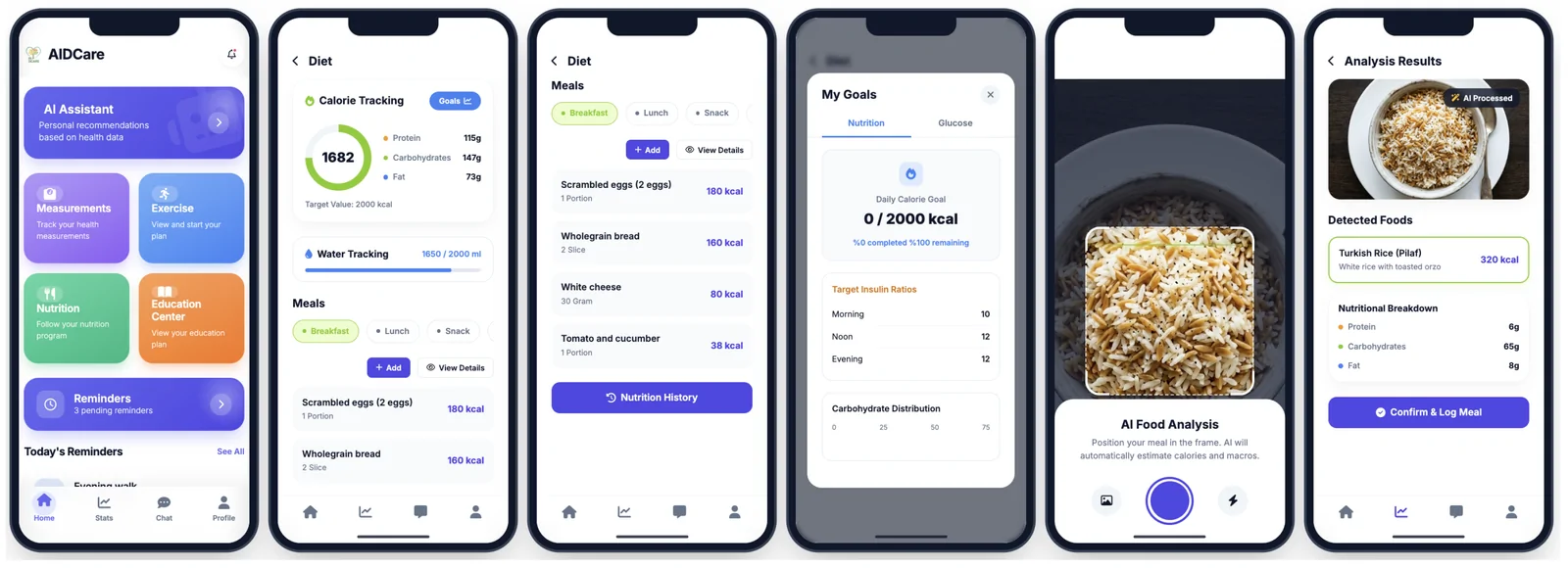
Implementation interface of the Nutrition and Diet Module within the AIDCare platform, illustrating the integration of camera-based food classification with structured nutritional information and daily dietary goals.

The Nutrition and Diet Module processes dietary information obtained through the AIDCare application. The module supports food classification and nutrient estimation, including carbohydrates, protein, and fiber. Camera-based food information can be transformed into structured nutritional information for subsequent processing. Figure 8 demonstrates the implementation interface of the Nutrition and Diet Module, highlighting how visual food classification is integrated with daily dietary goals to support comprehensive nutritional management.

### 4.2. Clinician Dashboard Implementation

A clinician-facing component was implemented to provide professional oversight of the patient and AI-assisted health-management workflow. The Clinician Dashboard provides access to relevant patient information and outputs generated by the AIDCare platform. The dashboard is connected to the backend infrastructure, allowing relevant information maintained within the patient record to be presented to the clinician.

The clinician-in-the-loop functionality allows healthcare professionals to review and rectify Medical LLM responses and manage patient-specific lifestyle goals and treatment-related information. This functionality introduces a human oversight layer into the AI-assisted workflow and provides a mechanism for correcting or adjusting system outputs when required.

**Figure 9:**
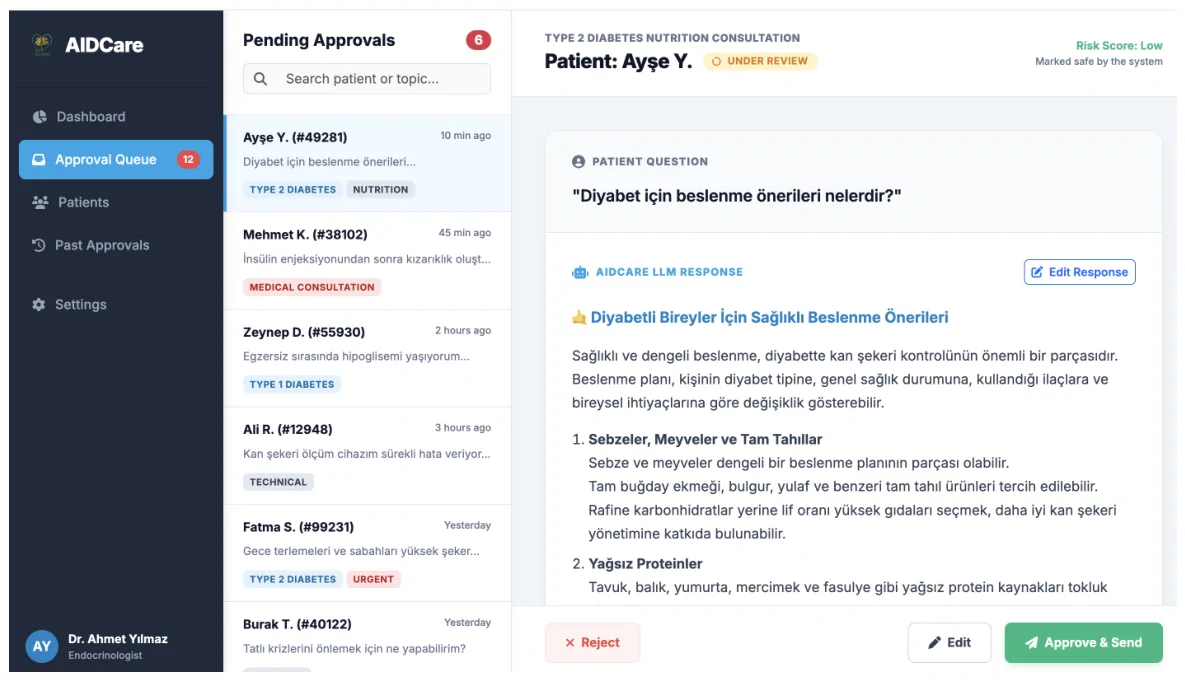
Implementation interface of the Clinician Dashboard and clinician-in-the-loop functionality within the AIDCare platform, illustrating the presentation of patient information, health-monitoring records, analytical outputs, personalized recommendations, and Medical LLM interactions within a clinician-facing environment.

The clinician interface therefore complements the automated components of AIDCare rather than replacing them. Sensor-derived information, health-analysis outputs, personalized recommendations, and Medical LLM interactions can be considered within a clinician-supervised environment. This design supports the intended use of AIDCare as a digital health-management platform and not as an autonomous clinical decision-making system. As illustrated in Figure 9, the Clinician Dashboard provides a comprehensive oversight environment where healthcare professionals can monitor patient progress and review AI-generated insights.

## 5. Results and Discussion

The implementation of AIDCare resulted in an integrated mHealth platform that connects multimodal smartphone sensing, IoMT-based wearable information, health monitoring, intelligent analysis, personalized interaction, medical content, and clinician oversight within a common application environment. The implemented system provides a patient-facing mobile application connected to backend services and a PostgreSQL database, allowing information acquired from different sources to be processed and maintained within a unified patient context.

The implemented architecture demonstrates that smartphone-based sensing can be incorporated into a broader diabetes self-management workflow rather than being used solely for isolated data collection. The smartphone camera, microphone, and accelerometer provide complementary input modalities, while the smartwatch extends the sensing layer with connected wearable information. These inputs are made available to the AIDCare backend and subsequently to the relevant health-analysis and personalization components.

The implementation therefore establishes the proposed sensing-to-application pathway in which the smartphone acts simultaneously as a user interface, sensing platform, and gateway to connected IoMT devices. This is particularly relevant to home- and community-based diabetes management because the sensing and interaction functions are integrated into a device that can be used during routine daily activities.

### 5.1. Multimodal Smartphone and IoMT Sensing

The implemented sensing layer integrates multiple sources of patient information through the AIDCare mobile application. The smartphone accelerometer provides activity-related information for the exercise-management component, while the camera provides visual input for the nutrition and diet workflow. The microphone provides an additional patient interaction modality, and smartwatch-derived information complements the smartphone-based measurements.

The integration of these modalities demonstrates the feasibility of establishing a multimodal sensing layer within a mobile diabetes-management application. Rather than requiring every category of information to be manually entered, AIDCare provides multiple mechanisms through which patient information can be acquired. The resulting information can subsequently be processed by specialized health-management modules.

To evaluate pipeline reliability systematically, 400 experimental trials (100 per modality) were executed under predefined failure thresholds across two reference mobile hardware ecosystems: (i) an Android testbed comprising a Samsung Galaxy A73 (Android 16) paired with a Samsung Galaxy Watch 5 (Wear OS 6 via BLE 5.0), and (ii) an iOS testbed comprising an Apple iPhone 17 Pro Max (iOS 27) paired with an Apple Watch Series 8 (watchOS 9.5 via BLE 5.0). For each sensing modality, 50 trials were conducted on Android and 50 on iOS. Testing was partitioned equally across two network environments: 200 trials conducted over local Wi-Fi and 200 trials over commercial cellular infrastructure, encompassing background app suspension, camera flash actuation, and simulated transient connection drops. An acquisition failure was defined as a hardware I/O initialization timeout exceeding 3 s or an empty sensor buffer. A transmission failure was designated as a BLE GATT disconnect event, HTTP socket timeout exceeding 5 s, or unrecovered network drop. A processing failure was classified as an unhandled backend exception, schema parsing mismatch, or JSON deserialization error during data normalization.

An important outcome of the implementation is therefore the integration of heterogeneous sensing modalities within a single application architecture. However, the present evaluation should be interpreted as an architectural and functional demonstration rather than a clinical-grade evaluation of sensor accuracy. The study did not evaluate the clinical-grade accuracy, sensitivity, specificity, energy consumption, or long-term reliability of the individual smartphone or wearable sensors. Such evaluation requires dedicated sensor-performance experiments under real-world conditions. The functional evaluation results for the multimodal sensing layer are presented in Table 2.

The results in Table 2 provide a functional view of the implemented sensing pipeline across acquisition, transmission, and processing stages. The smartphone camera and accelerometer demonstrate high functional completion across the evaluated workflow, while the microphone and smartwatch similarly demonstrate successful integration with the AIDCare application and backend. The overall results indicate that heterogeneous sensing inputs can be transferred through the application and made available to downstream processing components.

### 5.2. Health Monitoring and Analysis Functionality

The implemented AIDCare platform provides a set of interconnected health-monitoring and analysis modules covering physiological measurements, HbA1c-related risk assessment, insulin dosage recommendation, nutrition and diet assessment, and exercise analysis. The Vital Measurements Module provides the primary mechanism for recording and managing daily patient measurements, while the specialized analytical modules process information relevant to different aspects of diabetes self-management.

The Nutrition and Diet Module supports food classification and nutrient estimation, including carbohydrates, protein, and fiber. This functionality provides structured dietary information that can subsequently contribute to the broader patient context and the insulin-related calculation pathway. Similarly, the Exercise Module utilizes activity-related information obtained through the sensing layer to represent the patient’s physical activity.

The HbA1c Risk Prediction module and Insulin Dosage Recommendation Engine extend the platform beyond basic monitoring by transforming available patient information into higher-level analytical and decision-support outputs. These modules are integrated with the broader AIDCare architecture rather than functioning as independent applications. Their outputs can therefore contribute to personalized recommendations and clinician review.

The implementation demonstrates the feasibility of combining physiological, dietary, and activity-related information within a common digital health environment. A functional overview and integration outcome of each implemented monitoring and analysis component is summarized in Table 3. The principal result at this stage is the successful integration of heterogeneous health information into a common patient context, allowing information generated by different modules to be accessed by downstream personalization and clinician-facing components.

Nevertheless, the present study does not establish the clinical effectiveness of the prediction or insulin-related components. Their predictive accuracy, clinical safety, and utility require validation using appropriately designed clinical datasets and prospective studies. In particular, the insulin dosage recommendation functionality should be considered a technical decision-support component of the prototype rather than a clinically validated treatment recommendation.

### 5.3. Medical LLM and Personalized Interaction

The implementation also integrates a fine-tuned Medical Large Language Model (LLM) into the AIDCare platform. The Medical LLM provides a conversational interaction mechanism through which patients can interact with the system using their available health context. Patient conversation history is maintained within the platform, allowing subsequent interactions to retain relevant contextual information rather than treating each interaction as an isolated request.

To quantitatively assess recommendation relevance and domain adaptation, the evaluation task was formulated as a clinical intent classification and decision-support benchmark across four operational classes. Evaluation was conducted on the held-out test split of *n* = 2461 document-level educational articles, partitioned using random seed 42 and strictly sequestered from all training, prompt tuning, and model selection. The class distribution in the test set comprised: dietary adjustments (38.0%, n=935), physical activity guidance (26.0%, n=640), glycemic monitoring reminders (21.0%, n=517), and physician consultation prompts (15.0%, n=369). Precision, recall, and F1-score were computed using macro-averaging (the unweighted arithmetic mean across all four classes), ensuring that safety-critical but lower-frequency categories like physician consultation prompts receive equal clinical importance rather than being obscured by dominant lifestyle categories (for reference, the weighted-average F1-score was 81.60%).

Ground-truth clinical intent labels were verified across a representative subset of 300 multi-turn clinical scenarios independently evaluated in a blinded, randomized format by three healthcare professionals: an endocrinologist (>15 years of specialized diabetes care experience), a registered dietitian (>10 years in medical nutrition therapy), and a certified diabetes care and education specialist (>12 years in outpatient diabetes education). In 279 of the 300 cases (93.0%), all three clinicians agreed unanimously. For the remaining 21 cases (7.0%) where an initial two-to-one split occurred, disagreements were resolved via a structured consensus adjudication conference to establish unanimous ground-truth target labels. Inter-rater concordance prior to adjudication demonstrated strong reliability, yielding a multi-rater Fleiss’ κ=0.84 and an average pairwise Cohen’s κ=0.86. True positives, false positives, and false negatives were determined by exact-match category alignment of the model’s generated structured recommendation tokens against the adjudicated ground truth.

The held-out test set comprised 2461 document-level items, each assigned to one of the four predefined operational intent classes during benchmark construction: dietary adjustments, physical activity guidance, glycemic monitoring reminders, or physician consultation prompts. These benchmark-assigned labels were used as the reference labels for the full quantitative evaluation of accuracy, precision, recall, and F1-score. The remaining 2161 items were not independently adjudicated by clinicians. To assess the clinical plausibility of the benchmark labeling scheme, a representative subset of 300 multi-turn clinical scenarios was additionally reviewed by three healthcare professionals in a blinded and randomized manner. In 279 of the 300 cases (93.0%), all three clinicians agreed unanimously; the remaining 21 cases (7.0%) were resolved through structured consensus adjudication. Inter-rater concordance before adjudication yielded a Fleiss’ κ of 0.84 and an average pairwise Cohen’s κ of 0.86. Accordingly, the Fleiss’ κ and Cohen’s κ values apply only to the 300-item clinician-adjudicated subset and should not be interpreted as evidence that all 2461 test-item labels were independently clinician-validated. The complete 2461-item evaluation is therefore characterized as document-level intent classification on educational text rather than a clinician-validated assessment of recommendation quality. Model predictions were compared with the corresponding benchmark reference labels for the complete held-out test set using exact-match category alignment.

To establish comparative efficacy, performance was benchmarked against two baseline systems evaluated on the identical test split (*n* = 2461): (i) a classical TF-IDF feature extractor coupled with a multi-class Logistic Regression classifier, and (ii) the unadapted base MedGemma 4B-IT model operating in zero-shot mode without QLoRA fine-tuning. As shown in Table 4, the unadapted zero-shot base model achieved an accuracy of 62.40% and an F1-score of 61.10%, while the TF-IDF baseline achieved 68.20% accuracy and an F1-score of 67.50%. Domain-adapted QLoRA fine-tuning markedly improved performance, reaching 81.00% accuracy and an F1-score of 81.20% at epoch 20 (+20.1% F1 improvement over zero-shot base).

**Figure 10:**
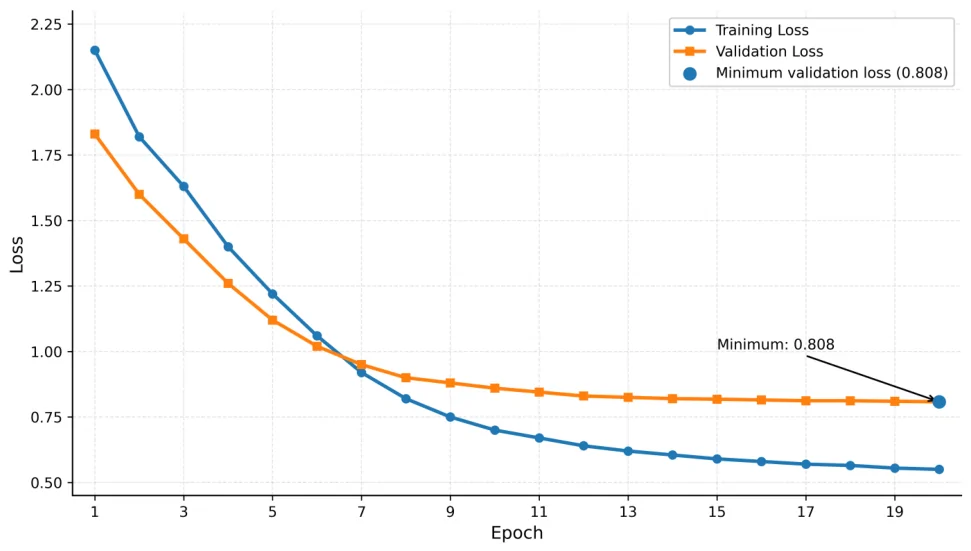
Cross-entropy training loss (*n* = 19,690) and validation loss (n=2461) trajectories across 20 QLoRA fine-tuning epochs on the normalized medical corpus. Validation loss plateaued at approximately 0.81 from epoch 12 onwards, indicating model capacity saturation as training completed at the scheduled 20 epochs.

Inspection of the loss trajectories in Figure 10 reveals that while training loss continuously declined from 2.15 to 0.55 across 20 epochs, validation loss reached an asymptote near 0.81 around epochs 12–15. This divergence indicates mild parameter overfitting and specialization to the stylistic patterns of hospital educational publications. Training was stopped at the scheduled epoch 20; validation loss had already plateaued around epochs 12–15.

The final precision of 83.5%, recall of 79.0%, and F1-score of 81.2% quantify the model’s document-level intent classification performance on the held-out educational-text evaluation set. These metrics should not be interpreted as evidence that the generated recommendations were clinically appropriate, safe, or effective. The clinician-adjudicated subset provides evidence of inter-rater agreement for the benchmark labeling approach, but it does not establish clinical recommendation quality across the full 2461-item test set. Clinical appropriateness, safety, and effectiveness of the generated recommendations require separate evaluation using clinically curated cases and independent healthcare-professional assessment.

The Medical LLM is connected to the wider AIDCare architecture and can operate alongside structured health information and patient-specific lifestyle goals. This integration allows conversational interaction to complement the structured outputs generated by the health-monitoring and analytical modules. Relevant information from physiological measurements, HbA1c-related risk assessment, nutrition and exercise analysis, and patient-specific goals can therefore contribute to the broader context available to the intelligent interaction component.

The AI Personalized Recommender further connects these components by utilizing information generated across the platform to provide individualized health-management support. Relevant information can include physiological measurements, HbA1c-related risk information, nutrition and exercise information, patient goals, and contextual information generated through Medical LLM interactions. This allows recommendations to be generated using a broader patient context rather than relying on a single health measurement or sensing modality.

The fine-tuning results provide quantitative evidence of the performance of the Medical LLM-based recommendation component under the adopted evaluation protocol, while the implemented integration demonstrates its ability to operate within the broader multimodal AIDCare architecture. The combination of conversational interaction, patient context, and personalized recommendations provides a mechanism for connecting smartphone- and IoMT-derived information with individualized diabetes self-management support.

### 5.4. Medical Content and Clinician-in-the-Loop Functionality

The AIDCare implementation incorporates structured medical content through the H5P Parsing Engine. H5P content is uploaded to the backend, where the implemented parser extracts the relevant content and converts it into a structured representation. The resulting information is subsequently normalized using Second Normal Form (2NF) before being stored and integrated into the AIDCare application. This process provides a structured pipeline for transforming interactive medical resources into machine-processable content that can be utilized by the intelligent components of the platform.

A total of 24,612 medical articles were obtained from the structured H5P medical content and used to construct the training corpus for fine-tuning the Medical LLM. The model selected for this purpose was MedGemma 4B-IT. Parameter-efficient fine-tuning was performed using Quantized Low-Rank Adaptation (QLoRA), which enables adaptation of the language model using low-rank trainable adapter parameters while maintaining the pretrained base model in a quantized form. This approach reduces the computational and memory requirements associated with full-parameter fine-tuning and is therefore suitable for resource-constrained GPU-based training environments.

The QLoRA-based fine-tuning process was performed using an NVIDIA RTX 5000 GPU. The structured medical content was transformed into the training format required by the Medical LLM and used to adapt the model toward the medical domain represented by the AIDCare medical-content corpus. In this configuration, the 24,612 medical educational articles served as the supervised fine-tuning corpus rather than as a vector database or embedding-based retrieval store. Thus, the purpose of the fine-tuning process was to adapt the model’s behavior and domain-specific response patterns, while the structured AIDCare database continued to provide patient-specific and application-level information.

The use of structured medical content establishes a direct connection between the medical-content layer and the Medical LLM component of AIDCare. Consequently, the medical content is not limited to patient-facing medical material but also contributes to the development of the domain-adapted conversational intelligence integrated into the platform.

Table 5 summarizes the medical-content processing and Medical LLM fine-tuning pipeline.

The QLoRA-fine-tuned Medical LLM is subsequently integrated into the wider AIDCare architecture. The model can operate alongside structured patient information, health-analysis outputs, patient-specific goals, and conversation history. This integration allows the domain-adapted language model to provide contextualized interaction while maintaining access to information generated by the other components of the platform.

Importantly, the fine-tuning process and retrieval of patient-specific information represent separate functions within the AIDCare architecture. Fine-tuning is used to adapt the pretrained MedGemma 4B-IT model to the domain represented by the medical-content corpus, whereas structured patient information and application data provide instance-specific context during interaction. Consequently, an embedding model or vector database such as ChromaDB is not inherently required for the QLoRA fine-tuning process itself.

The clinician-facing component provides an additional layer of oversight for the AI-assisted workflow. The Clinician Dashboard allows relevant patient information and AI-generated outputs to be reviewed, while the Clinician-in-the-Loop module supports correction of Medical LLM responses and management of patient-specific goals and treatment-related information. This mechanism ensures that AI-generated information is positioned within a supervised healthcare workflow rather than being treated as an autonomous clinical decision.

The combination of domain-specific Medical LLM fine-tuning, multimodal patient information, personalized interaction, and clinician oversight represents an integrated approach to intelligent diabetes self-management. The corpus of 24,612 medical educational articles provides the training foundation for domain adaptation of the Medical LLM, while the AIDCare architecture provides the patient-specific context and interaction mechanisms required for personalized support.

### 5.5. Quantitative Evaluation of Personalized Recommendation

**Figure 11:**
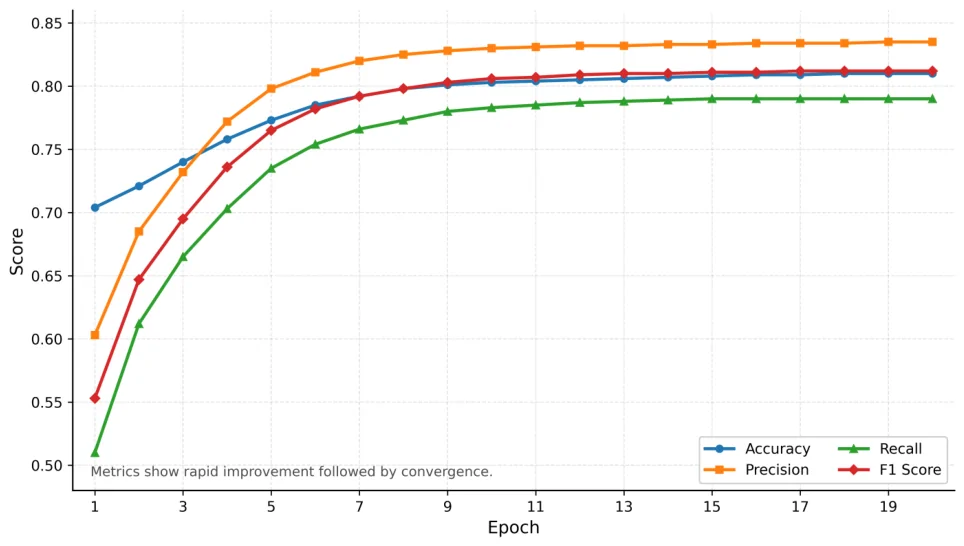
Progression of evaluation metrics (accuracy, precision, recall, and F1-score in %) on the held-out validation split (n=2461) across 20 QLoRA fine-tuning epochs, demonstrating convergence toward an F1-score of 81.2%.

The quantitative evaluation focused on the performance of the personalized recommendation component following fine-tuning of the Medical LLM on the AIDCare medical-content corpus. The fine-tuning process was monitored over 20 epochs using training loss, validation loss, accuracy, precision, recall, and F1-score. The training and validation loss curves are presented in Figure 10, while the progression of the performance metrics is presented in Figure 11.

Figure 10 shows a progressive reduction in training and validation loss during the fine-tuning process. The observed convergence indicates that the model progressively adapted to the domain-specific medical content used during training. The training and validation curves remained relatively consistent during the later epochs, suggesting stable optimization under the adopted fine-tuning configuration.

The progression of accuracy, precision, recall, and F1-score is presented in Figure 11. The performance measures increased during the initial stages of fine-tuning and subsequently approached a relatively stable level. The final recorded performance reached an accuracy of approximately 81.0%, precision of 83.5%, recall of 79.0%, and an F1-score of 81.2%.

As detailed in Table 4, domain-adapted QLoRA fine-tuning enabled the Medical LLM to achieve a final accuracy of 81.0%, precision of 83.5%, recall of 79.0%, and an F1-score of 81.2% across the held-out test split (n=2461). This represents a marked improvement over both the unadapted zero-shot MedGemma 4B-IT baseline (F1: 61.1%) and the classical TF-IDF classifier (F1: 67.5%).

The final precision of 83.5% indicates that a substantial proportion of the recommendations identified as relevant by the system were consistent with the reference decisions used during evaluation. The recall of 79.0% indicates that the system recovered a substantial proportion of the relevant recommendation instances. The resulting F1-score of 81.2% provides a combined measure of precision and recall and represents the principal performance indicator for the personalized recommendation component.

The observed convergence across the training process, together with the final performance metrics, demonstrates the technical feasibility of adapting the Medical LLM to the domain-specific medical content incorporated within AIDCare. The results also indicate that the fine-tuned model can support the personalized recommendation functionality integrated into the broader platform.

However, the reported metrics should be interpreted within the scope of the underlying evaluation protocol. They should not be interpreted as evidence of clinical effectiveness, clinical safety, or improved patient outcomes. In particular, the fine-tuning results demonstrate model performance within the available development and evaluation setting but do not establish how the model would perform across diverse real-world patient populations or unseen clinical scenarios.

Further evaluation using an independently held-out clinical evaluation dataset, clinically curated test cases, and assessment by healthcare professionals is required to establish the generalizability, factual reliability, safety, and clinical utility of the Medical LLM-based recommendation component.

### 5.6. Discussion

The results demonstrate the technical feasibility of integrating multimodal smartphone sensing, wearable information, image-based nutrition and diet assessment, physical activity analysis, Medical LLM interaction, personalized recommendation, structured medical content, and clinician oversight within a unified diabetes self-management platform. By bridging daily dietary intake, continuous physiological feedback, and clinician-guided artificial intelligence, the platform establishes an automated assessment-to-personalization pathway for structured diabetes lifestyle management. The functional system evaluation achieved overall acquisition, transmission, and processing success rates of 97.5%, 96.5%, and 95.5%, respectively, across 400 experimental trials, while the fine-tuned Medical LLM achieved an F1-score of 81.2% on structured clinical recommendation intents with an average backend response latency under 1.8 s. Together, these findings indicate that heterogeneous digital health modalities can be successfully harmonized within a single mobile architecture under controlled conditions.

A primary operational advantage of AIDCare is the transformation of heterogeneous patient information into structured clinical and nutritional telemetry. Rather than requiring users to manually navigate disparate calorie-counting apps, fitness trackers, and blood glucose logs, the platform centralizes data acquisition and processing within a unified patient context. Image-based meal captures are converted into estimated carbohydrate, protein, and fiber masses, which directly inform prandial insulin bolus calculation and personalized dietary scoring. Simultaneously, accelerometer data provides active energy expenditure metrics that contextualize glycemic dynamics. This multi-domain integration is particularly relevant to diabetes self-management, where dietary habits, physical exertion, and physiological status are fundamentally interdependent. Similar to recent findings by Gackowski et al. [51] highlighting the importance of coordinated digital platforms for bridging patient self-management with professional care plans, AIDCare demonstrates that embedding clinical oversight directly into AI-driven lifestyle architectures provides a viable blueprint for continuous, supervised chronic disease support.

#### 5.6.1. Significance for Digital Nutrition and Precision Lifestyle Management

A critical question in digital health is how technological architectures contribute directly to nutritional science. In diabetes care, Medical Nutrition Therapy (MNT) is recognized as the foundational pillar of metabolic control. However, conventional nutritional methodology has long been hindered by the limitations of self-reported dietary recalls and food frequency questionnaires, which frequently suffer from 20% to 50% underreporting of energy and carbohydrate intake, severe portion misestimation, and high patient fatigue. By integrating camera-based food recognition with volumetric approximation and reference food composition databases (USDA FoodData Central and TÜRKOMP), AIDCare advances the “sensors-to-nutrients” paradigm. It converts meal photography into quantitative macronutrient telemetry—specifically estimating carbohydrates, protein, and dietary fiber.

Furthermore, nutritional intake does not occur in a physiological vacuum. Identical meals provoke markedly different postprandial glycemic excursions depending on co-ingested dietary fiber, meal protein content, prior physical exertion, and baseline insulin sensitivity. AIDCare closes this metabolic loop by linking real-time image-based nutrient quantification directly with wearable sensor feedback and clinical decision support.

#### 5.6.2. Translational Research Roadmap and System Refinement

While the current study demonstrates system-level technical feasibility, substantial further research is required to refine, validate, and translate AIDCare into clinical practice. Because the present evaluation was conducted as an engineering proof-of-concept using synthetic patient profiles and controlled software pipelines, a comprehensive five-phase translational research roadmap is outlined below:

1. **Analytical Validation of Image-Based Nutrient Estimation:** The computer-vision dietary module must be rigorously benchmarked against gold-standard weighed food records, duplicate meal chemical analyses, and standardized food-image databases. Critical nutritional challenges—such as estimating mixed composite dishes, detecting hidden fats and added sugars, handling visual occlusion in layered foods, and accounting for diverse cultural food preparations—require quantitative determination of Mean Absolute Error (MAE) and Mean Absolute Percentage Error (MAPE) for carbohydrate, protein, and fiber estimation.
2. **Closed-Loop Physiological Glycemic Validation:** Prospective observational studies pairing AIDCare meal captures with continuous glucose monitoring (CGM) are needed to validate the relationship between estimated macronutrient metrics (e.g., net carbohydrates and fiber-to-carbohydrate ratios) and actual postprandial glucose excursions across diverse metabolic phenotypes.
3. **Human-Centered Usability and Ergonomic Refinement:** Extensive usability testing utilizing standardized instruments (System Usability Scale [SUS], NASA-TLX) must be conducted with diverse patient cohorts—stratified by diabetes duration, age, and digital health literacy—as well as multidisciplinary clinical teams (endocrinologists, registered dietitians, and diabetes educators) to minimize cognitive burden and prevent long-term tracking attrition.
4. **Prospective Randomized Controlled Trials (RCTs):** Rigorous clinical trials in individuals living with Type 1, Type 2, and gestational diabetes are essential to determine whether using AIDCare produces measurable clinical improvements in primary metabolic endpoints, including glycated hemoglobin (HbA1c) reduction, Time-in-Range (TIR: 70–180 mg/dL), reduction in glycemic variability ($\sigma_{G}$), and incidence of severe hypoglycemia, alongside sustained behavioral adherence to nutritional goals.
5. **Clinical Safety Guardrails and Regulatory Compliance:** Automated decision-support algorithms—particularly insulin bolus calculations and LLM-generated recommendations—require redundant clinical safety guardrails, including strict maximum bolus caps, mandatory hypoglycemia lockouts, active insulin-on-board subtraction, and formal certification under Software as a Medical Device (SaMD) regulatory frameworks prior to real-world deployment.

## 6. Conclusions

This paper presented the design, implementation, and system-level evaluation of AIDCare, an integrated mHealth platform that connects camera-based nutritional assessment, wearable activity tracking, physiological monitoring, and clinician-supervised conversational AI for diabetes self-management. The architecture establishes an automated assessment-to-personalization pathway that links everyday dietary macronutrient intake (carbohydrates, protein, and dietary fiber) and physical activity with structured health analysis and professional clinical oversight.

System-level evaluation demonstrated the technical viability of the platform. Across 400 experimental software-pipeline trials, AIDCare achieved data acquisition, transmission, and processing completion rates of 97.5%, 96.5%, and 95.5%, respectively, while serving backend inferences with an average response time under 1.8 s. The fine-tuned Medical LLM achieved 81.0% accuracy, 83.5% precision, 79.0% recall, and an F1-score of 81.2% on a structured clinical recommendation intent benchmark. Importantly, the clinician-in-the-loop dashboard ensures that automated nutrient and activity interpretations remain under professional medical supervision, allowing healthcare providers to adjust personalized recommendation parameters.

AIDCare demonstrates the technical feasibility of bridging digital nutritional methodology, wearable sensor telemetry, and intelligent decision support into a cohesive diabetes lifestyle management framework. Because the present evaluation was established as a laboratory proof-of-concept using synthetic profiles and pipeline testing, future work must execute the outlined translational roadmap—validating nutritional estimation accuracy against weighed dietary records, evaluating real-world usability, and conducting prospective clinical trials to establish long-term metabolic efficacy and safety.

## Figures and Tables

**Table 1:** Feature comparison of leading clinical diabetes digital health platforms and the proposed AIDCare mHealth Platform.

Feature	mySugr	Glooko	One Drop	LibreView	AIDCare
*Data acquisition and sensing integration*					
Manual blood glucose and vital logging	√	√	√	√	√
Manual dietary and carbohydrate logging	√	√	√	×	√
Camera-based automated nutrient estimation	×	×	×	×	√
Wearable physical activity sync	√	√	√	×	√
Multimodal sensing pipeline	×	×	×	×	√
*Clinical decision support and analysis*					
Prandial insulin bolus calculation	√	×	×	×	√
Longitudinal HbA1c risk estimation	√	√	√	√	∘
Active energy expenditure estimation	×	×	×	×	√
Contextual multi-objective recommender	×	×	∘	×	∘
*Intelligent interaction and clinical oversight*					
Domain-adapted Medical LLM conversational agent	×	×	×	×	√
Clinician-facing oversight dashboard	√	√	√	√	√
Clinician-in-the-loop parameter weighting	×	×	×	×	√
Structured medical educational corpus integration	×	×	×	×	√

**Note:** √ denotes implementation and system/pipeline-level evaluation, not clinical accuracy validation. Camera-based nutrient estimation and prandial bolus calculation were implemented but not accuracy-validated. The symbol ∘ denotes an architected or preliminary prototype function while the symbol × means that there is no implementation of the corresponding feature.

**Table 2:** Evaluation of multimodal data acquisition, transmission, and backend ingestion pipeline completion across 400 bench testing trials. Rates reflect software pipeline success [n/N] with 95% Wilson binomial confidence intervals [53], not clinical sensor measurement accuracy.

Sensing Modality	Trials (*N*)	Acquisition Completion [*n/N*] (%, 95% CI)	Transmission Completion [*n/N*] (%, 95% CI)	Backend Processing [*n/N*] (%, 95% CI)	Primary Pipeline Function
Accelerometer	100	98/100 (98.0%, 93.0–99.4)	97/100 (97.0%, 91.5–99.0)	96/100 (96.0%, 90.2–98.4)	Physical activity and exercise assessment
Smartphone Camera	100	99/100 (99.0%, 94.6–99.8)	98/100 (98.0%, 93.0–99.4)	97/100 (97.0%, 91.5–99.0)	Food image acquisition and nutrition assessment
Microphone	100	97/100 (97.0%, 91.5–99.0)	96/100 (96.0%, 90.2–98.4)	95/100 (95.0%, 88.8–97.8)	Voice-based patient interaction
Smartwatch	100	96/100 (96.0%, 90.2–98.4)	95/100 (95.0%, 88.8–97.8)	94/100 (94.0%, 87.6–97.2)	Wearable physiological and activity information
**Overall**	**400**	**390/400 (97.5%, 95.4–98.6)**	**386/400 (96.5%, 94.2–97.9)**	**382/400 (95.5%, 93.0–97.1)**	**Multimodal AIDCare sensing pipeline**

**Table 3:** Summary of implemented health-monitoring and analysis functionality in AIDCare.

Module	Input Information	Implemented Functionality	Integration Outcome
Vital Measurements	Patient-recorded physiological measurements and available sensor-derived health information	Recording, management, and longitudinal organization of daily patient measurements	Provides structured health information for downstream analysis, personalization, and clinician review
HbA1c Risk Prediction	Relevant patient physiological measurements	HbA1c-related risk assessment based on available patient information	Prediction output is incorporated into the common patient context and made available to downstream components
Insulin Dosage Recommendation	Patient information and nutritional information, particularly estimated carbohydrate content	Technical insulin dosage recommendation based on available patient context	Provides decision-support output within the clinician-supervised architecture
Nutrition and Diet	Patient-generated dietary information and camera-based food information	Food classification and estimation of carbohydrates, protein, and fiber	Produces structured dietary information for patient context and insulin-related processing
Exercise and Activity	Smartphone accelerometer and connected smartwatch activity information	Representation and assessment of patient physical activity	Provides activity-related information for integration with broader diabetes self-management context
**Overall Integration**	**Physiological, dietary, and activity-related information**	**Interconnected health monitoring and analysis within a common platform**	**Supports downstream personalization and clinician-facing review**

**Table 4:** Clinical intent classification and recommendation performance on the held-out test split (n=2461) comparing baseline models against the fine-tuned Medical LLM across selected training epochs.

Model/Configuration	Training Loss	Validation Loss	Accuracy (%)	Precision (%)	Recall (%)	F1-Score (%)
***Baseline Comparators* (*Held-out Test Set*, *n* = 2461)**						
TF-IDF + Logistic Regression	—	—	68.20	70.50	64.80	67.50
Base MedGemma 4B-IT (Zero-shot)	—	—	62.40	64.10	58.50	61.10
***Fine-Tuned MedGemma 4B-IT with QLoRA* (*n* = 2461)**						
Epoch 1	2.15	1.83	70.40	60.30	50.90	55.30
Epoch 5	1.22	1.12	77.30	79.80	73.50	76.50
Epoch 10	0.70	0.86	80.30	83.00	78.30	80.60
Epoch 15	0.59	0.82	80.80	83.30	79.00	81.10
**Epoch 20 (Final)**	**0.55**	**0.81**	**81.00**	**83.50**	**79.00**	**81.20**

**Table 5:** Medical content processing and Medical LLM fine-tuning pipeline.

Component	Implementation	Role in AIDCare
Medical content source	H5P-based medical content	Provides domain-specific medical and diabetes-related knowledge for the platform.
Content processing	Custom H5P Parsing Engine	Extracts and converts H5P content into a structured representation.
Data normalization	Second Normal Form (2NF)	Organizes the extracted content and reduces unnecessary data redundancy.
Medical content corpus	24,612 medical articles	Provides the supervised training corpus for Medical LLM fine-tuning.
Medical LLM	MedGemma 4B-IT	Provides the pretrained language-model foundation for domain adaptation and conversational interaction.
Fine-tuning method	QLoRA	Enables parameter-efficient adaptation of the Medical LLM with reduced computational and memory requirements.
Training hardware	NVIDIA RTX 5000 GPU	Provides the GPU-based computational environment used for QLoRA fine-tuning.
Training integration	H5P-derived medical content → QLoRA fine-tuning → Medical LLM	Adapts the language model to the medical content represented in the AIDCare corpus.
Personalization context	Patient information, conversation history, and patient-specific goals set by respective clinician	Provides contextual information for individualized interaction and recommendations.
Clinical oversight	Clinician Dashboard and Clinician-in-the-Loop module	Enables professional review and correction of AI-generated outputs and management of patient-specific information.

## Data Availability

The data and research materials supporting the findings of this study are available from the corresponding author upon reasonable academic request through the author’s institutional email address. Access may be provided for academic validation and research purposes, subject to applicable institutional and data-use requirements.

## Abbreviations

    The following abbreviations are used in this manuscript:

AI	Artificial Intelligence
AIDCare	AI-assisted Diabetes Care
API	Application Programming Interface
BLE	Bluetooth Low Energy
BMI	Body Mass Index
GPU	Graphics Processing Unit
H5P	HTML5 Package
HbA1c	Hemoglobin A1c
ICR	Insulin-to-Carbohydrate Ratio
IoMT	Internet of Medical Things
ISF	Insulin Sensitivity Factor
JSON	JavaScript Object Notation
LLM	Large Language Model
MET	Metabolic Equivalent of Task
mHealth	Mobile Health
QLoRA	Quantized Low-Rank Adaptation
REST	Representational State Transfer
UI	User Interface
